# A singular value decomposition approach for improved taxonomic classification of biological sequences

**DOI:** 10.1186/1471-2164-12-S4-S11

**Published:** 2011-12-22

**Authors:** Anderson R Santos, Marcos A Santos, Jan Baumbach, John A McCulloch, Guilherme C Oliveira, Artur Silva, Anderson Miyoshi, Vasco Azevedo

**Affiliations:** 1 Department of General Biology, Instituto de Ciências Biológicas, Universidade Federal de Minas Gerais, Belo Horizonte, Av. Antônio Carlos, 6627, MG, 31.270-901, Brazil; 2 Computer Science Departament, Instituto de Ciências Exatas, Universidade Federal de Minas Gerais, Belo Horizonte, Av. Antonio Carlos, 6627, 31.270-901, MG, Brazil; 3 Max Planck Institute for Informatics, Campus E2 1, Saarbrücken, Germany; 4 CEBio and Laboratory of Cellular and Molecular Parasitology, Instituto René Rachou, Oswaldo Cruz Foundation, Belo Horizonte, Av. Augusto de Lima 1715, 30190-002, MG, Brazil; 5 Genome and Proteome Network of the State of Pará, Universidade Federal do Pará, Belém, R. Augusto Corrêa, 66.075-110, PA, Brazil

## Abstract

**Background:**

Singular value decomposition (SVD) is a powerful technique for information retrieval; it helps uncover relationships between elements that are not *prima facie* related. SVD was initially developed to reduce the time needed for information retrieval and analysis of very large data sets in the complex internet environment. Since information retrieval from large-scale genome and proteome data sets has a similar level of complexity, SVD-based methods could also facilitate data analysis in this research area.

**Results:**

We found that SVD applied to amino acid sequences demonstrates relationships and provides a basis for producing clusters and cladograms, demonstrating evolutionary relatedness of species that correlates well with Linnaean taxonomy. The choice of a reasonable number of singular values is crucial for SVD-based studies. We found that fewer singular values are needed to produce biologically significant clusters when SVD is employed. Subsequently, we developed a method to determine the lowest number of singular values and fewest clusters needed to guarantee biological significance; this system was developed and validated by comparison with Linnaean taxonomic classification.

**Conclusions:**

By using SVD, we can reduce uncertainty concerning the appropriate rank value necessary to perform accurate information retrieval analyses. In tests, clusters that we developed with SVD perfectly matched what was expected based on Linnaean taxonomy.

## Background

We developed a methodology, based on singular value decomposition (SVD), for improved inference of evolutionary relationships between amino acid sequences of different species [1]. SVD produces a revised distance matrix for a set of related elements. Our SVD-based computations provide results that are close to the internationally accepted scientific gold standard, Linnaean taxonomy.

The reason we chose this methodology is the proven capacity that SVD has to establish non-obvious, relevant relationships among clustered elements [2][3][4][5], providing a deterministic method for grouping related species. A distance matrix derived from SVD can be used by cladogram software to produce a "phylogenetic tree", yielding a visual overview of the relationships. We compared species grouping by this method with Linnaean taxonomy grouping and found that the species clusters were similar.

The rationale behind SVD is that a matrix A can be represented by a set of derived matrices [2], in the same way that a number can be derived into factors. One can also think of SVD as a set of matrices that provide numerically different representations of data without loss in semantic meaning, as for example representation in different base numbers. To understand the mathematical concept of SVD, suppose that 'A' is an array of real numbers or complex numbers composed of m rows by n columns. A matrix with a singular value decomposition of matrix A can be made:(1)

where U is an orthonormal m x m matrix, and Σ is an m x n matrix, known as the diagonal matrix, with real and non negative numbers. The matrix *V^T^* is known as a conjugate transpose, an n x n unit matrix with real or complex numbers. As the diagonal values of Σ are ordered in descending order, Σ is a direct function of matrix A and characterizes the singular values of this matrix, ordering them from the most significant to the least significant values. Considering a subset of singular values of size k<n, we can obtain *A_k_* an approximate matrix of matrix A:(2)

Thus, data approximation depends on how many singular values are used [6]. In this case, the number of singular values k is also known as the rank of matrix *A_k_*, indicating how many lines and columns in matrix *A_k_* are linearly independent. The possibility of extracting information based on less data is part of the reason for this technique’s success, as it allows data compression/decompression, with an execution time that does not increase exponentially with increasing matrix size, making analysis viable [6]. A data set represented by a smaller number of singular values than the original, full-size data set has a tendency to group data items that would not be grouped together if we used the original data set [2]. This could explain why clusters derived from SVD can expose non-trivial relationships among the original data set items [7]. In this paper we do not use the matrix *A_k_*, product's factorization by SVD to rank k; with only two arrays of SVD, the matrix *D_k_*[3] is represented in the context of the matrix(3)

The justification for using only *D_k_* is that it has k lines instead of m lines from *A_k_*, so *D_k_* is made up of linear combinations from *U_k_* columns, which in turns provides the relationship *A* ≈ *A_k_* ≈ *D_k_*.

**Figure 1:**
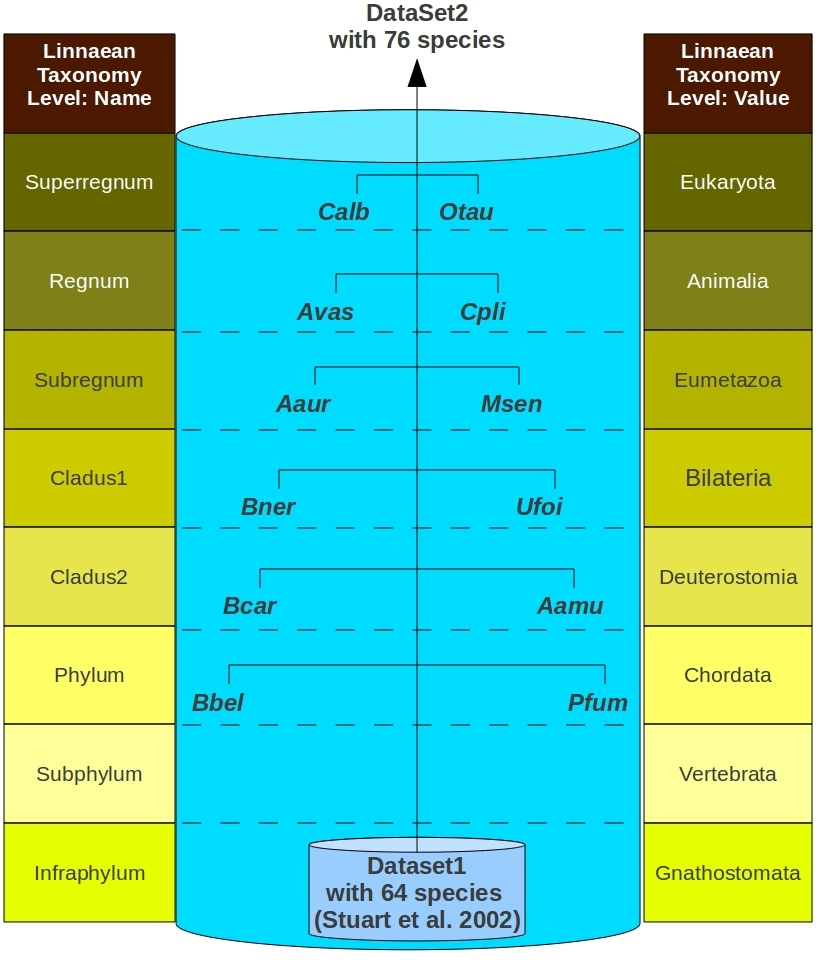
**Dataset2 schema.** Construction scheme for a set of species that were used as a negative control for the partitioning techniques.

The main data set that we used was obtained from a previous study involving SVD [8], with 832 mitochondrial protein sequences from 13 families of mitochondrial genes, obtained from 64 vertebrate mitochondrial genomes. We organized these 832 sequences into 64 single FASTA sequences, each representing a single Linnaean species, concatenating the sequences of the 13 families of mitochondrial genes of each species. From here on, we will refer to this set of data as dataset1. Dataset1 consists of 64 highly-related species that have at least 8 of 14 Linnaean taxonomy levels in common with each other. As we also wanted to investigate how SVD parameters can influence cluster quality, we added 12 additional species to this data set, creating a second set of data, which we named dataset2 (Figure 1). We chose these 12 new species based on their high diversity, in order to create a less homogeneous data set; our objective was to determine whether SVD would separate non-related and related species into different groups. The species within dataset1 all belong to the same infraphylum (*Gnathostomata*), whereas the 12 new species that were included to increase diversity were selected from other phyla, also from the animal kingdom. The 12 species included in dataset2 were *Aphrocallistes vastus*, *Asterias amurensis*, *Aurelia aurita*, *Balanoglossus carnosus*, *Branchiostoma belcheri*, *Bugula neritina*, *Callyspongia plicifera*, *Candida albicans*, *Metridium senile*, *Ostreococcus tauri*, *Phallusia fumigata*, and *Unionicola foili.*

The quality of the clusters that were generated was measured by the number of Linnaean taxonomy levels each species within the cluster bore in common with the other species; this was calculated as a function of an increasing rank value. When certain rank values are reached, larger values do not improve cluster quality, because there is no increase in taxonomic levels that the species have in common; in some cases a decrease is observed. The cluster quality obtained from a certain rank value maintains the number of shared common Linnaean taxonomy levels constant. This is evidence that there is an intrinsic relationship between these species that is mirrored in the distance matrix derived from these clusters; this quality helps build relevant cladograms.

## Results and discussion

### Singular value decomposition and number of clusters matters

In this study we give support to the hypothesis that choice of an appropriate data representation and a fixed number of clusters, combined with a good algorithm for categorizing this data, is sufficient for the production of biologically significant clusters. An A matrix has rank n, where n indicates the number of distinct species. The rank value (k) defines the degree of resolution of matrix *D_k_* compared to the original matrix D, so k must be less than or equal to n. However, a k value close to n is undesirable, because one obtains a strong approximation to the original matrix D, which is useless to uncover relationships. We need to avoid this so-called ‘noise data’ [9] and find a smaller number of singular values that adequately represent the original data and thus achieve a reduction in the amount of data that needs to be processed [9][10]. We found that there is an optimal rank value that can be obtained by systematically testing all possible rank values and distances that define whether two species will form part of the same cluster, based on Linnaean taxonomic levels. A maximum distance value defining whether two species belong to a cluster can be experimentally found by increasing and decreasing an initial, empirically-defined distance, for example, the maximum distance between two species in a data set. We tried a systematic search for parameters that could confirm or deny this hypothesis. Working with singular value decomposition, one of the main parameters is the number of singular values necessary to create matrix decomposition sufficient to correctly separate all 76 species. This can be done by an algorithm called kdcSearch, which systematically examines possibilities for variation in singular values, Euclidean distance separation of clusters and number of clusters, a triad that we call kdc values. A systematic search to evaluate these three parameters proved to be computationally viable, independent of human intervention; it separates the target species into groups that represent similarity relationships between protein sequences and thus infer homology between species. The clusters generated through systematic choice of these parameters were biologically significant, demonstrating that we were on the right path in our attempt to determine the smallest number of singular values and the correct Euclidean distance that will correctly represent the original data, giving the correct separation of species groups. Based on these experiments we showed that even an “as simple as possible agglomerative clustering algorithm” (ASAP) can benefit from singular value decomposition to improve the quality of clusters that are generated. The next step was to use the parameters that were optimal [5] according to our methodology in other algorithms that have been thoroughly tested by the scientific community. The choice was made by K-Means [11], Expectation Maximization (EM) [12], Adaptive Quality-based Clustering Algorithm (AQBC) [13], K-Medoids [14], and MakeDensityBasedClusterer (MDBC) [15], since there is a statistically well-founded background, they have been widely used, and they are available as free software packages from R [16], Waikato Environment for Knowledge Analysis (WEKA) [15], and the JAVA Machine Learning Library [17]. The K-Means requires that an array of numbers be processed to calculate distances for the creation of clusters. It also opens the possibility of including a parameter that defines a fixed number of clusters to be created with the elements in the distance matrix. The same number of clusters inferred from the analysis done by ASAP, our in-house agglomerative clustering algorithm, was used by the K-Means algorithm. The K-Means implemented in the R statistical software, from now on called the K-Means-R algorithm, was parameterized for the initial number of elements, but not for specific elements. There is no such parameterizing in the K-Means implemented in the WEKA (K-Means-WEKA) software, making it possible that different results will be obtained with these two programs. We chose as the number of initial elements for calculating the first K-Means-R average half of the items or half of the species. The first run of K-Means-R was done with a matrix regarded as adequate because it had been generated with the parameters of the algorithm systematically observed ASAP, a rank value of nine and eight clusters. The algorithms EM, MBDC, K-Means-WEKA and K-Medoids were configured for eight clusters, without altering the other configuration parameters. The algorithm AQBC does not allow fixing the number of clusters, but we empirically tested parameters till we obtained the same number of clusters (eight). Then we looked for a way to compare the results from the various algorithms. At first glance it seemed that the result of, for example, K-Means-R was as good as the result from ASAP, but the large number of species and the not less considerable number of clusters made the comparison difficult. We needed a measure that would allow us to objectively compare the performances of the algorithms. Then we initiated execution of all algorithms with a number of singular values that represented the original array, without any reduction in the rank of the matrix decomposed into singular values. Despite minor variations in quality in some clusters, the overall quality of the clusters did not differ from the performance of all algorithms on a distance matrix generated with a reduction in rank. Table 1 shows quality calculations of eight clusters using ASAP and K-Means-R algorithms with different numbers of singular values. Clusters shown in this table are from the second round of trying to create smaller clusters, while maintaining correct separation of the *Aves* group (positive control), or the first recursive call of the ASAP algorithm. Both K-Means-R and ASAP were configured to generate eight clusters. Both algorithms used the matrix of the trigrams representing 8,000 combinatorial possibilities of 20 amino acids (20^3^), also called N-gram with N=3, with 60 singular values (the original matrix, since all possible singular values were used) and another matrix derived from the former with only nine singular values; these quantities of singular values and clusters gave good SVD results in final clustering. The first column shows the cluster identification. The columns that follow are in groups of four, showing the results of K-Means and ASAP, using a trigrams frequency matrix created by SVD with 60 or nine singular values. The four columns under the label 'Number of species clusters Joined by' show the number of species obtained in each cluster. The four columns under the label 'Linnaean Taxonomy levels in common by clusters' show the number of Linnaean levels in common for each cluster, and the four columns under the label 'common Linnaean taxonomy frequency levels (cLtlf) by cluster' show the results of the metrics that we suggested. These come from multiplication of the column 'Number of species clusters Joined by' by the column 'Linnaean Taxonomy levels in common by clusters'. There were no significant differences (Student t test) in the quality of clusters generated by the algorithms, based on a comparison of the mean number of Linnaean levels in common and cLtlf, even though they used different singular values, as shown in the Additional file 1 (Figure S1). The Chi-square test did not demonstrate any significant relation between these four clustering rounds. However, there were significant differences between the algorithms and cluster data, based on cLtlf, as shown in Table 2. This table shows an alternative to measuring algorithm performance with different calibration parameters, using Linnaean taxonomy to infer cluster quality. The sum of the individual qualities of each cluster is measured by cLtlf. When cLtlf is weighted by the variation of this quality around the mean or standard deviation, the quality of results can be inferred through Linnaean clusters metric quality (Lcq). It is worth noting that clusters whose data are shown in Table 1 possessed a large number of taxonomic levels in common (60% of the levels that we used in this work). It is possible that so many in-common taxonomic levels left little scope for differentiation between the clusters, making the average quality very similar regardless of the method used for clustering. This result in the comparison between K-Means-R and ASAP was also observed in the results produced by the other algorithms that we tested. When there was a set of clusters with homogeneous qualities, it was necessary to find a measure that would discriminate the effectiveness of the algorithms with different numbers of singular values. Therefore, we used a measurement that takes into consideration the sum of the qualities of all clusters provided by a given method, weighted by the variation in the quality of clusters. We analyzed this metric to look for significant differences between the two algorithms and the two numbers of singular values. The K-Means-R algorithm performance was two-fold better than that of ASAP. When we used an array of nine decomposed singular values, the number considered optimal for this set of data, in accordance with the methodology suggested here, K-Means and ASAP algorithms had 9 and 28% better performances, respectively, when compared to the original results from these methods, without singular value decomposition. The other algorithms that we tested also gave a significant increase in the quality of clusters in the results of the matrix decomposed into nine singular values and eight clusters, versus the non-decomposed matrix and eight clusters. In decreasing order, the increases in performance for each method were ~50% (AQBC), ~49% (EM), ~27% (ASAP), ~16% (K-Medoids), and ~9% (K-Means-WEKA, MDBC and K-Means-R). Despite the equal percentage increase for the algorithms K-Means-WEKA, MDBC and K-Means-R, the absolute quality values for K-Means-R were approximately 50% higher than those from K-Means-WEKA and MDBC, considering the distance matrices with and without decomposition by singular values. We chose the K-Means-R method for more detailed analysis of the results because this is a widely used algorithm and because in terms of absolute quality, it gave results very close to those from algorithm EM, which was the best in terms of absolute quality. These results have some details that are worthy of note. First, they show that in fact a matrix decomposed into a certain number of singular values, using a certain number of clusters, can create a representation of the original data with better quality than that obtained when we use the original data matrix (full rank). This reinforces the need for decomposition of a matrix into a smaller number of singular values for the removal of so-called 'noise' attributable to a full-rank array [9][10][18]. Second, the clustering algorithm was instrumental in generating good-quality clusters. It can be seen in Table 2 that the performance of K-Means-R and EM algorithms was two-fold better than that of the ASAP algorithm. Third, the method that we suggest here, to systematically explore the parameters needed to obtain the best performance of the K-Means proved essential to allow the K-Means to generate even better quality clusters. Fourth, the representation of a sequence of amino acids as a vector that stores the trigram frequency of 20 amino acids was effective to capture the levels of similarity between the sequences of the protein species that we analyzed, without incurring the problems that classical algorithms have with protein sequence alignments [19]. Fifth and finally, the quality metrics using the Linnaean classification suggested in this study were effective in measuring the quality of the biological significance of clusters constructed from mitochondrial proteins of dozens of species. Consequently, we conclude that when we use a smaller number of singular values to generate clusters, the quality of the clusters is significantly improved when compared with clusters generated with a matrix with all singular values, independently of algorithm. These results show that the combination of correct choice of algorithm, the number of singular values, the number of clusters and a quality metric with biological significance allows separation of species groups that are biologically meaningful. Furthermore, the use of trigrams of amino acids provides an effective way to determine similarity between protein sequences without using sequence alignment algorithms.

**Table 1:** Using the distance matrix that corrected separated *Aves* cluster: K-Means compared to ASAP

	Number of species joined by clusters				Linnaean Taxonomy levels in common by clusters				common Linnaean taxonomy levels frequency (cLtlf) by cluster			
**Cluster**	**K-means with rank 60**	**SNJ with rank 60**	**K-means with rank 09**	**SNJ with rank 09**	**K-means with rank 60**	**SNJ with rank 60**	**K-means with rank 09**	**SNJ with rank 09**	**K-means with rank 60**	**SNJ with rank 60**	**K-means with rank 09**	**SNJ with rank 09**

**1**	10	10	10	10	10	10	10	10	100	100	100	100
**2**	14	27	14	25	10	9	10	9	140	243	140	225
**3**	4	1	4	7	12	13	12	8	48	13	48	56
**4**	7	17	4	7	8	8	10	8	56	136	40	56
**5**	2	2	9	2	13	11	9	12	26	22	81	24
**6**	6	1	4	4	10	13	10	10	60	13	40	40
**7**	5	1	6	4	9	13	10	12	45	13	60	48
**8**	11	1	8	1	9	13	8	13	99	13	64	13

This table displays the results of K-Means and ASAP on a cluster of 60 species obtained in the first ASAP clustering round, when 76 species were separated into clusters.

**Table 2:** Inferring quality from clustering methods

Algorithm/ software	Rank	N	Min cLtlf	Max cLtlf	Mean cLtlf	cLtlf clusters sum (∑cLtlf)	cLtlf standard deviation (σ)	Linnaean clusters quality (∑cLtlf/σ)	Linnaean clusters quality gain (K09/K60)%	cLtlf median	Median clusters quality gain (K09/K60)%
AQBC-javaml	K09	8	32	180	71.25	570	52.27	10.90	49.58%	42.50	26.87%
				
	K60	8	0	220	64.38	515	70.64	7.29		33.50	

EM-weka	K09	8	40	120	70.12	561	31.53	17.79	48.99%	57.00	1.79%
				
	K60	8	16	160	70.25	562	47.06	11.94		56.00	

Kmeans-weka	K09	8	30	180	69.38	555	46.70	11.88	9.26%	61.50	-2.38%
				
	K60	8	16	180	69.88	559	51.39	10.88		63.00	

Kmeans-R	K09	8	40	140	71.62	573	34.48	16.62	9.21%	62.00	6.90%
				
	K60	8	26	140	71.75	574	37.72	15.22		58.00	

K-Medoids-R	K09	8	24	160	70.12	561	44.37	12.64	15.92%	60.00	13.21%
			
	K60	8	26	180	68.50	548	50.24	10.91		53.00	

MDBC-weka	K09	8	30	180	69.38	555	46.70	11.88	9.26%	61.50	-2.38%
				
	K60	8	16	180	69.88	559	51.39	10.88		63.00	

ASAP-in house	K09	8	13	225	70.25	562	67.68	8.30	27.51%	52.00	197.14%
				
	K60	8	13	243	69.12	553	84.92	6.51		17.50	

All evaluated partitioning's algorithms showed improved performance considering the Linnaean clusters quality when used the optimized distance matrix created by the better kdc parameters tested.

In the remainder of this paper, we show preliminary findings and methods that helped us reach our final conclusions, including how we arrived at an adequate number of singular values that allowed us to separate a set of species into groups with biological significance. To this end, we found that using arrays of trigram frequencies of amino acids to determine statistical properties was as good as using 4-gram frequencies [19]. We show that the size of the sequences that are analyzed can affect the separation of elements into clusters. We also present measures that allow us to infer the biological significance of a cluster and measure the quality of the clustering methods compared to Linnaean taxonomic classification of species.

### Algorithm kdcSearch: parameterizing rank and number of partitions

**Figure 2:**
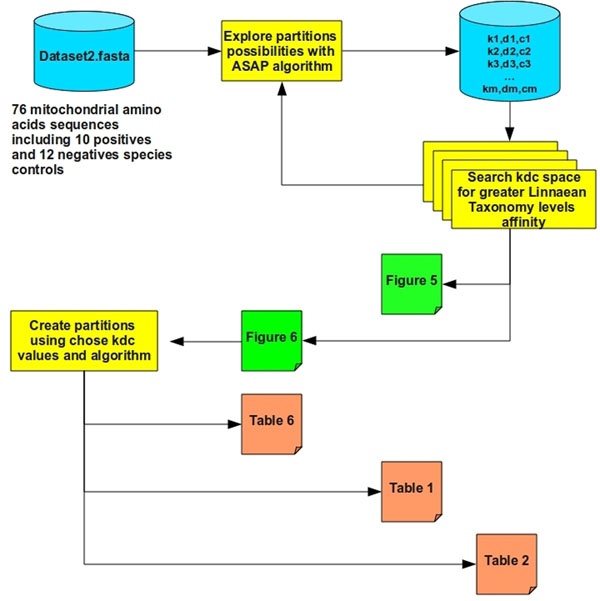
**kdcSearch algorithm schema.** Main procedures, datasets and products. Multiple rectangles mean recurring calls.

The objective of the algorithm kdcSearch (Figure 2) is to identify a 'k' rank value and a quantity 'c' of partitions that promote correct separation of species, based on biological significance according to Linnaean taxonomy. This 'k' rank is responsible for the reducing the dimensions of the data that hide evolutionary relations among species, also known as data noise. A quantity of partitions 'c' should correctly separate the positive control group from the other species and possibly separate the other species into partitions with evolutionarily-significant relationships. In this algorithm, the number of partitions 'c' is a function of 'd', that is c=f(d), with 'd' being the Euclidian distance between elements in a symmetric matrix of distances between the species. The value of 'd', on the other hand, varies according to the distance matrix created with rank 'k', establishing the relations d=f(k) and c=f(f(k)). In this way, we look for the 'k' value that will eliminate data noise and generate a distance 'd' responsible for creating a number of partitions 'c' with the greatest capacity to infer evolutionary relationships between grouped species. In this process, we use an in-house algorithm, ASAP, to partition the species. Considering the random selection of pivotal elements for the creation of partitions by ASAP, it is not possible to estimate a priori what distance 'd' will create a number 'c' of partitions. Consequently, a systematic search is made with a range of X and Y values of Euclidean distances in order to determine which distances give what number of partitions. This algorithm begins with an X value equal to the largest Euclidean distance between species represented in a symmetric matrix of distances with a maximum value; that is, it has not undergone decomposition by singular values and for this reason k is equal to the total 'n' of the number of species in this matrix. Considering the maximum distance of the symmetric matrix, only one partition with 'n' species is formed. The value Y is always zero, the point at which the search for partitions of value 'k' terminates and 'n' partitions of the data are always formed, with only one species per partition. With the values in hand (k1, k2, k3, ..., kn) with their respective values (c1=f(d1) , c2=f(d2), c3=f(d3), ..., cn=f(dn)), in turn with their respective biological significance levels, measured by the function cLtlf, we filtered the configuration that gave the correct separation of the control group and the greatest number of partitions of species sharing the highest possible numbers of Linnaean classification levels. This algorithm is recursive because if no group of these variables provides a partitioning of the control group isolated from the other species, then the algorithm did not yet find a solution with the desired level of Linnaean taxonomic relationship. In order to simplify, it is not necessary to analyze all of the possible numbers of partitions; analysis is made only within well-defined intervals. Taking as an example, dataset2 with 76 species, an alternative is to analyze the number of partitions containing groups of three (c3, c6, c9, ..., c75). This example can be obtained from the algorithm below through the initialization of a variable that, as it divides the total number of species by 25, permits the creation of an incremental step of three levels between analyses. The number was determined empirically and the algorithm below is adduced by the variable EDRD (Empirical Dimensional Range Division).

When one of the recursions of the algorithm kdcSearch finds one or more groups of variables k, d and c that give correct separation of the positive control group, the algorithm recursions are finalized. In this case, there is no reason to continue making recursions, since the desired level of cohesion for the elements of the partitions has reached its limit, measured by the positioning of the positive control. In the case of the data that we analyzed here, this situation occurs after the end of the first recursion by the algorithm kdcSearch, culminating in the plotting of the final graphs and implementing the function 'Finalize'. The code for the function 'Finalize' was left open because at this stage of execution, the algorithm finds various groups of the variables k, d and c (kdc) that promote correct separation of the positive control group in a partition separate from the other species. At this point, the question is which group of values kdc is a good result. What differentiates one group of variables kdc from another is the quality of the partitioning of the other species compared with Linnaean taxonomic classification. We think that it would not be useful to develop an algorithm that one particular kdc group is better than others because they give different levels of separation of species. A researcher can be trying to separate a group of species at the level of 'Classis' with nine Linnaean levels in common (Table 3), while another researcher may try to separate this same group at the 'Ordo' level, with 11 Linnaean levels in common. Consequently, it would be reasonable to consult the last table generated by the algorithm kdcSearch to adjust the result to the necessities of a specific objective. However, in case the final objective is not well defined, an option to completely automate this process could be to compare the partitioning medians for each kdc group with which it was possible to separate integrally and isolatedly the positive control species group. This comparison creates an estimate of the cohesiveness of the partitionings based on comparison with Linnaean taxonomic classifications. Values of kdc that give larger medians would be chosen as superior, promoting partitionings with greater biological significance. The rationale that explains the use of the median as a parameter for the procedure 'Finalize' can be better comprehended by analysis of the data in Tables 4 and 5. These tables show the cLtlf results for nine sets of kdc values that by definition are good results because they can separate integrally and isolatedly the positive control group. In the partitionings produced with these kdc values, there is always a partition of the positive control group with a cLtlf equal 100 (10 species sharing 10 Linnaean levels). Values of kdc that cannot optimally separate the positive control group from the other species were also included. The set of kdc values used as a negative control in this analysis is suffixed with the symbol '(-)'. In Table 4, we can see that the kdc sets that have many partitions with only one isolated element (cLtlf=1*13=13 or the minimum cLtlf) reduce the median cLtlf value for all of the partitions produced in this set by the respective set of kdc values. The intention of these partitionings is to demonstrate evolutionary relationships among species; the kdc values that give large numbers of partitions with only one element each do not give much information about such relationships. Consequently, it is understood that the best kdc values are those that have the fewest species isolated in partitions with only one element. Table 5 also shows the application of the measurement 'Linnaean cluster quality' to the partitionings based on these kdc values; however, this measure was not effective in indicating how informative the partitionings for each group of kdc values were in terms of the relationships based on Linnaean classification. It can be seen that the kdc values of the negative control had larger 'Linnaean cluster quality' values than the various sets of kdc values that adequately separated the positive control group. Apparently, 'Linnaean cluster quality' is not efficient at classifying kdc values at this 'Finalize' step of the algorithm search, though it is efficient while the positive control group has not been integrally separated in an isolated partition. However, based on the median, the sets of kdc values that do not separate the positive control group into isolated clusters were correctly classified as being of low quality based on Linnaean classification, as well as other kdc values that had many partitions with the lowest Ltlf. In Table 5, the kdc values with the largest medians are in bold, and the kdc values that do not adequately separate the positive control group are in italic. It is relevant to point out that though some kdc values can adequately separate the positive control group, many partitions have the minimum cLtlf; these were responsible for the low kdc values, values even lower than some kdc values that do not adequately separate the positive control group. In this study, we decided to analyze in more detail the partitions created by the kdc values with eight partitions and rank nine, which produced the third best median result without separating many species into isolated partitions. This choice is justified by the fact that these kdc parameters make the correct separation of the mammals 'Hsap' and 'Ppya' in a partition separate from those of the other species. These two species were used as a second positive control group. In the set of kdc values with six partitions and rank six, the configuration classified as having the best median, these two species are in a partition with 25 other species. Another option would be to use the kdc values with six partitions and rank three, which were classified as the second-best median. In this kdc configuration, 'Hsap' is isolated in a partition, while 'Ppya' is in a partition with 26 other species. Accordingly, the kdc values that give eight partitions with rank nine promote correct separation of the two positive control groups and were responsible for significantly improving performance in the statistic 'Linnaean cluster quality' and in most of the medians of the partitioning algorithms that were tested (Table 2).

**Table 3:** Linnaean taxonomy levels

Linnaean Taxonomy levels		
**Number**	**Name**	**Value**

14	*Species*	*Aythya americana*
13	*Genus*	*Aythya*
12	*Familia*	*Anatidae*
11	*Ordo*	*Anseriformes*
10	*Subclassis*	*Carinatae*
9	*Classis*	*Aves*
8	*Infraphylum*	*Gnathostomata*
7	*Subphylum*	*Vertebrata*
6	*Phylum*	*Chordata*
5	*Cladus2*	*Deuterostomia*
4	*Cladus1*	*Bilateria*
3	*Subregnum*	*Eumetazoa*
2	*Regnum*	*Animalia*
1	*Superregnum*	*Eukaryota*

Linnaean taxonomy levels used to classify the species in this paper. The numbers denote an increasing degree of nomenclature specialization.

**Table 4:** Function Finalize: sample data

06clusters k03	06clusters k06	08clusters k06	08clusters k09	*08clusters k12(-)*	08clusters k45	*10clusters k30(-)*	12clusters k12	14clusters k18	14clusters k21	*14clusters k36(-)*	14clusters k60
**100**	**100**	100	**100**	*248*	100	*144*	100	100	100	*88*	100
**243**	**243**	200	**225**	*13*	243	*252*	216	240	250	*240*	220
**45**	**64**	56	**56**	*180*	13	*13*	13	13	13	*13*	13
**96**	**100**	13	**56**	*30*	136	*13*	64	90	88	*96*	112
**13**	**13**	100	**24**	*13*	22	*56*	22	22	22	*22*	22
**40**	**40**	45	**40**	*32*	13	*13*	13	13	13	*20*	20
	
		24	**48**	*13*	13	*13*	16	16	13	*13*	13
		40	**13**	*13*	13	*13*	24	24	13	*13*	24
		
						*13*	40	40	30	*13*	13
						*13*	48	13	13	*13*	13
		
							13	13	13	*13*	13
							13	13	13	*13*	13
		
								13	13	*13*	13
								13	13	*13*	13

The statistic cLtlf for all of the partitionings of species obtained with nine kdc values that separate the positive control group in the function Finalize of the algorithm kdsSearch, along with three kdc values as a negative control (-).

**Table 5:** Function Finalize: sample statistics

ASAP/ Clusters	Rank	N	Min cLtlf	Max cLtlf	Mean cLtlf	cLtlf clusters sum (ΣcLtlf)	cLtlf standard deviation (σ)	Linnaean clusters quality (ΣcLtlf/σ)	cLtlf median
**06clusters**	**K03**	**6**	**13**	**243**	**89.50**	**537**	**82.46**	**6.51**	**70.50**
**06clusters**	**K06**	**6**	**13**	**243**	**93.33**	**560**	**80.81**	**6.93**	**82.00**
08clusters	K06	8	13	200	72.25	578	60.65	9.53	50.50
**08clusters**	**K09**	**8**	**13**	**225**	**70.25**	**562**	**67.68**	**8.30**	**52.00**
*08clusters(-)*	*K12*	*8*	*13*	*248*	*67.75*	*542*	*92.41*	*5.87*	*21.50*
08clusters	K45	8	13	243	69.12	553	84.92	6.51	17.50
*10clusters(-)*	*K30*	*10*	*13*	*252*	*54.30*	*543*	*81.02*	*6.70*	*13.00*
12clusters	K12	12	13	216	48.50	582	59.10	9.85	23.00
14clusters	K18	14	13	240	44.50	623	63.29	9.84	14.50
14clusters	K21	14	13	250	43.36	607	66.12	9.18	13.00
*14clusters(-)*	*K36*	*14*	*13*	*240*	*41.64*	*583*	*63.66*	*9.16*	*13.00*
14clusters	K60	14	13	220	43.00	602	60.68	9.92	13.00

Comparison of the Lcq values and the ΣcLtlf medians for partitionings of the species obtained in Table 4.

### From 76 to 60 species and eight clusters

**Figure 3:**
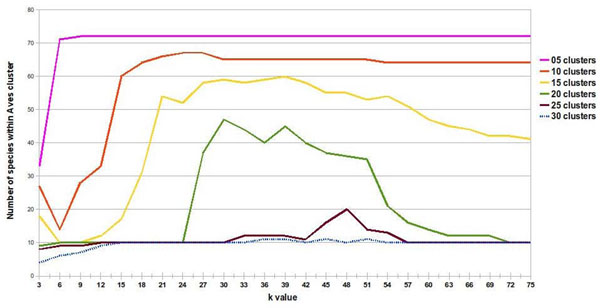
**Exploring the number of species in the *Aves* cluster.** The number of species grouped into the Aves cluster as a function of rank value and number of clusters. Ordinates are multiplied by the respective maximum Linnaean taxonomy levels shared by species in Figure 5.

**Figure 4:**
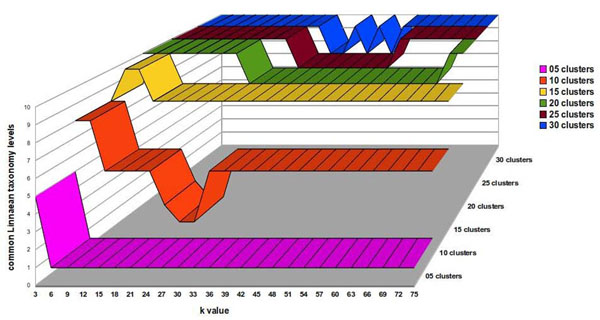
**Exploring *Aves* cluster with maximum shared linnaean taxonomy levels.** The number of Linnaean levels shared by all species is plotted against rank value and number of imposed clusters. Ordinates are multiplied by the respective number of species that produced Figure 5.

We decided to use a 76 species data set (dataset2), incorporating 12 species that were less related to the original group, in order to develop relationship trees that included clusters with distantly related species. The 64 species data set (dataset1) from the study by Stuart contains closely related species, as all of them share 8 of the 13 Linnaean taxonomy levels used in our study to differentiate species [5]. When a correct fit was made (15 imposed clusters and rank value of 39), we were able to separate 60 of the 64 species and the additional 12 species using ASAP (Figures 3 and 4). These 12 added species plus four of the original species from dataset1 did not group into a single cluster. Instead, we obtained several different clusters, most of which included only one species.

**Figure 5:**
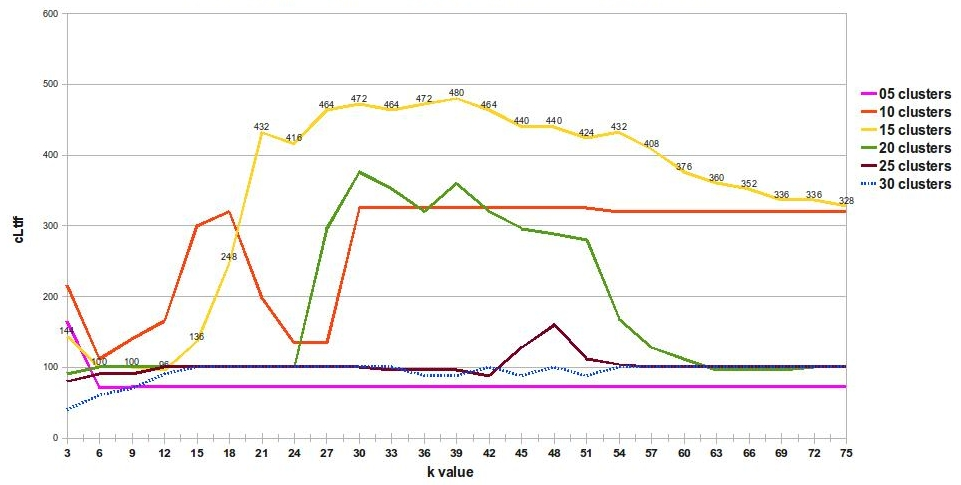
**Determining the best algorithm parameters.** Aves cluster quality as a function of rank value and different numbers of clusters. The number of clustered elements multiplied by maximum common Linnaean taxonomy levels shared between species gives the quality measure.

Analyses were then carried out on only the 60 species from the data set that were joined as a single cluster; the ASAP algorithm was run with 15 clusters and a rank value of 39. When the ASAP algorithm was run with the original 64 species data set, some elements were separated into isolated clusters despite actually sharing several Linnaean taxonomy levels in common with all of the other species.

**Figure 6:**
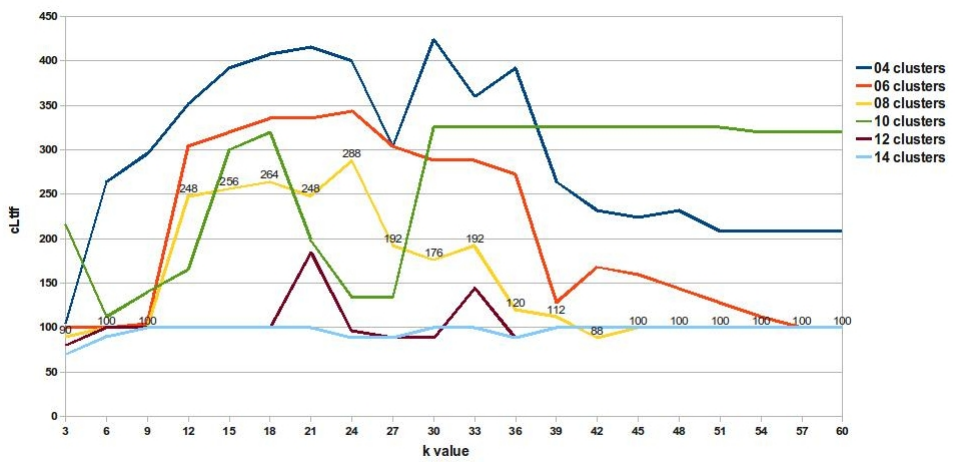
**Determining the best algorithm parameters at the first algorithm recurrence step.** Aves cluster quality measured with a reduced numbered of species than in dataset2. Now is possible to cluster the Aves species separately and the best algorithm adjustment to this cluster is preferred. Higher curves do not represent better quality.

**Figure 7:**
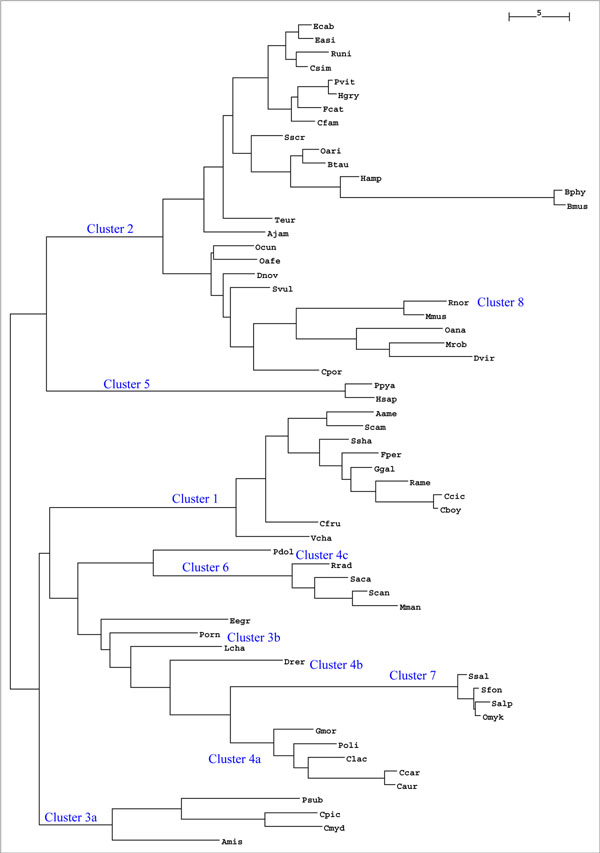
**60 species from the Stuart data set.** A 60 species data set unrooted tree generated from a distance matrix created with the ASAP algorithm. The original algorithm from this paper provided the distance matrix. Blue labels denote clusters.

This could be due to the fact that mitochondrial protein sequences for some species within the data set used in this study were not available. Since our algorithm only uses the frequency of occurrence of amino acid triplets, a lower frequency can affect the quality of the clusters that are generated, as does the presence or absence of a triplet sequence. Presence or absence of amino acid triplets are also responsible for early cluster separation of the 12 additional taxonomically distantly related species, incorporated into the original 64 species data set. Consequently, we worked with this 60 species data subset. To do so, we included a recurrence step prediction in our algorithm in order to develop a species subset. We worked with the concept that a good separation of species in clusters distributes the elements in groups of more than one element, whereas a group with only one element gives no information about species ancestry. When we correctly separated the *Aves* group in an isolated cluster, we assumed that other groups should also be close to divisions that have evolutionary significance. Finally, a good separation involves having *Aves* isolated in a single group, while having the largest possible number of other species together in groups, with a few isolated species in groups of only one element (Figure 5, rank value of 39 and 15 clusters). This definition of good separation between species is applicable only when it is not possible to isolate the positive control group, the *Aves* group. But when we split a homogeneous positive control group, the concept of a good separation of species is altered and it changes the way we interpret the graph of rank value versus cLtlf in the first recursive call of the algorithm (Figure 6, rank value of nine and eight clusters). In Figure 6, a high cLtlf value means poor cluster quality, because at this level of recursion it is possible to isolate the positive control group in a single cluster and leave few species in isolated groups. Therefore the optimal value for the separation of the group of *Aves* is 100 (10 species * 10 Linnaean levels in common). A value larger or smaller than that gave inappropriate separation, because the positive control is the group of birds with 10 species sharing 10 levels of Linnaeus. Using a second positive control group ('*Hsap*' and '*Ppya*'), we concluded that using eight partitions with rank nine is the best configuration, correctly separating the birds group, creating groups with evolutionary significance and decreasing the number of species in groups of only one element. We used a rank value of nine to create the unrooted tree shown in Figure 7. ASAP was calibrated with a d value that produces eight clusters using the experimented rank value of nine. This choice was made based on obtaining a good separation result, when grouping all species of the *Aves* class into a single cluster, plus a positive control group.

The results of the first execution of our recurrence algorithm based on the 60 species data set can be seen in Table 6. Clusters 2, 5 and 8 are comprised of species of theclass *Mammalia*. Cluster number 5 includes the hominids *Homo sapiens* and *Pongo pygmaeus*, which were together, separated from other mammals due to their mitochondrial protein sequences sharing 12 common Linnaean taxonomy levels. Clusters 2, 5 and 8 were composed of only mammalian species, sharing 9, 12 and 13 common Linnaean taxonomy levels, respectively. It is evident that the number of clusters and rank value used to create distance matrices enables even ASAP to provide adequate clustering based on quality discrimination. All the clusters that were obtained are shown in Figure 7, in which four mnemonic letters represent each species.

**Table 6:** Eight clusters from 60 data set

Cluster	Number of species joined	Linnaean taxonomy levels in common	Deepest Linnaean taxonomy level
1	10	10	*Carinatae*
2	25	9	*Mammalia*
3	7	8	*Gnathostomata*
4	7	8	*Gnathostomata*
5	2	12	*Hominidae*
6	4	10	*Elasmobranchii*
7	4	12	*Salmonidae*
8	1	13	*Rattus*

Eight clusters created from the first recurrence algorithm execution calibrated with a rank value of nine. Species were grouped according to their deepest evolutionary relatedness based on Linnaean taxonomy levels. Clusters 2, 5 and 8 belong to the mammalian class.

## Conclusions

Clusters and cladistic trees drawn from distance matrices, which were generated with SVD, showed a good correlation with Linnaean taxonomy. Considering the best estimate, when a difference is found, this does not necessarily mean strong divergence from taxonomic methods, but perhaps a more accurate picture of the relationship between the species that clustered together. This was demonstrated by clusters that were separated from mammalian clusters due to their greater protein sequence relatedness. It also was reinforced by Linnaean taxonomy information.

The similarity between clusters generated by our distance matrix and Linnaean taxonomy is indicative that distance matrices generated by SVD can demonstrate evolutionary relationships of species and construct better quality clusters and phylogenetic trees. These clusters and phylogenetic trees would benefit from amino acid trigrams and the Euclidean distance property of displaying a distance proportional to the number of necessary edits needed to perform a global alignment sequence within a polynomial execution time.

## Methods

### Datasets

The set of species used in this work is not original [8]. We opted for using a previously known set of data to allow comparisons with other studies that also use this data. We named this set of 13 mitochondrial proteins from 64 vertebrate species, dataset1. Within dataset1, a group of 10 species belonging to the class *Aves* was chosen to be the positive control group. We developed a negative control group with mitochondrial protein from 12 other species. Joining the proteins from these 12 species with the 64 in dataset1 gave origin to dataset2. Figure 1 schematically represents dataset2 as a set of data composted of dataset1 and 12 additional species. These 12 additional species were selected based on the criterion of being at least one level above the Linnean level common to all of the species in dataset1. Two species were randomly selected for each Linnaean taxonomic level, from *Phylum* to *Superregnum.* The same 13 mitochondrial proteins from dataset1 were selected for these 12 additional species. The additional amino acid sequences were obtained from the NCBI site. The union of these 13 mitochondrial proteins from the 12 new species with the sequences in dataset1 gave origin to dataset2, which includes positive and negative control groups of species. In order for a partitioning method to be successful, the positive control group needs to stay together in a partition and no other partition can be contaminated by the negative control group.

### Positive control group and statistics

In order to show how rank values and the number of imposed clusters affect SVD, we ran ASAP algorithm with different rank values and numbers of clusters. Figure 5 shows the results of these runs for a single cluster, the cluster denominated cluster 1, which contains species belonging to the Linnaean taxon, the *Aves* class. This taxon is ideal for testing our hypothesis, because few and closely related species within the data we used belonged to this taxon. Furthermore, the *Aves* species in our data set tended to mix with less evolutionarily related species when the algorithm was incorrectly calibrated or the number of clusters was too small. For evaluating the quality of the cluster generated, we considered the product of common shared Linnaean taxa among clustered elements multiplied by the number of clustered elements. This indicator gives us a good measure of cluster quality, as it assesses the frequency of commonality within the cluster. Here, we denominated this indicator as “common Linnaean taxonomy level frequency”, or cLtlf, and used it to show how cluster quality can vary as a function of the rank value or the maximum number of clusters used. Figure 5 shows the quality of cluster 1 generated by the algorithm, as rank value increases when different numbers of clusters are used to group the entire 76 species data set.

Figure 5 shows that, independent of the maximum number of clusters chosen to represent the 76 species data set, an increase in rank value does not improve cluster quality; consequently, we can safely use a considerably smaller number of singular values than the theoretical maximum. It is possible to roughly estimate an optimal value for rank value from this particular data set. If we consider 15 clusters, a rank value over 39 will not dramatically increase the quality of each cluster (Figure 5).

When we evaluate cluster quality measured by cLtlf, (Figure 5), we see that there is no significant improvement in cluster quality beyond the rank value of 39. This rank is sufficient for a good data representation of our original data set. Also, within cluster 1, the number of elements clustered together and the number of Linnaean taxonomy levels in common as a function of rank value, can be seen, respectively, in Figures 6 and 7. The maximum number of Linnaean taxonomy levels in common within cluster 1 obtained was 10. There is another interpretation for this graph in Table 3, associating these 10 levels in common within the cluster with the 14 Linnaean taxonomy levels considered in our study. This shows that the stringency of the data representation provided with SVD is sufficient to infer Linnaean taxonomy levels. On the other hand, if a less stringent fit is used, such as with an inappropriate number of clusters and rank value, a panoply of unrelated species are included in a cluster. It must be pointed out that our main task in this study was to learn and exemplify the calibration of our algorithm in order to retrieve desirable information. With the data set we used, the desirable information to be retrieved was Linnaean taxa, however, with other data sets this calibration should be tuned to direct the desired objective.

Table 3 characterizes a bird species, *Aythya americana*. The taxonomy levels shared by cluster 1 species in our algorithm executions with 20, 25, and 30 clusters and rank value 24, are levels lower than level number 11, namely the order (Ordo). Levels numbered as 11 (order) and 12 (family) were not shared among the 10 bird species in the data set. As more non-Aves species are added to this bird set, there is a decrease in cluster quality.

### Euclidean distance

We can produce a distance matrix that contains a measure of how each species is related to each other. To construct this matrix, each species rank values set is treated as a vector in a k-dimension space. One can choose the best measure to calculate the distance among vectors, depending on the particular characteristics in a data set. We decided to use Euclidean distance instead of the cosine distance used by Stuart [8]. This is because there is data indicating that Euclidean distance produces better cluster quality results than cosine distance. There is evidence [20], using the same 64 species data set that we present here, that Euclidean distance is proportional to the number of editions needed to perform a global sequence alignment. Consequently, it gives a more accurate measure of evolutionary relatedness than cosine distance, without the need for a global alignment sequence. There is evidence that the superiority of this Euclidean distance calculation is due to intrinsic evolutionary differences that affect the size of vectors. This is easy to see when one considers two vectors with the same cosine distance but with significant differences in length.

### ASAP algorithm: in house agglomerative clustering

We implemented a clustering algorithm that was called ASAP (As Simple As Possible) and showed that even a naive algorithm can benefit from data adequately treated by SVD. Thus, it is not our intention to demonstrate it's worth using this clustering algorithm, but we want to leave the message that regardless of the algorithm, it is worth using SVD conjugated with positive controls in information retrieval, as an initial filter against noise [10][18].

ASAP is an algorithm designed to facilitate the work of measuring the impact of using SVD in clustering algorithms. This algorithm somewhat resembles single-linkage clustering; the differences are that no clustering starts from the two elements with the lowest Euclidean distance. Clustering starts with a random element; also, a new entry is not inserted in the matrix of Euclidean distances for each cluster created between the algorithm interactions.

The idea is quite simple; randomly select a species from the distance matrix, cluster together with other species according to a fixed 'd' distance and remove the clustered species from the distance matrix. Do it again randomly selecting other species, and so on.

(1) Repeat as long as the number of columns in the distance matrix is greater than one:

1.1. Fix the first column as the pivotal element;

1.2. Create a cluster of elements so that the Euclidean distance is smaller than a 'd' value for the pivotal element;

1.3. Remove elements from the novel cluster (lines and columns) from the distance matrix;

1.4 End repeat.

This algorithm was implemented using Scilab1 5.2.1 run on GNU linux Ubuntu, core 2.6.22-16. This implementation is available in the Additional file 2, accompanied with data and raw results.

### Clustering algorithms evaluated

#### K-Means-R

The K-Means algorithm implemented [11] in the R statistical software aims to partition points into k groups such that the sum of squares from points to the assigned cluster centers is minimized. At the minimum, all cluster centers are at the mean of the set of data points which are nearest to the cluster center [16].

#### K-Means-WEKA

The K-Means algorithm implemented in the WEKA software is denominated SimpleKMeans. This implementation can use either the Euclidean distance or the Manhattan distance. If the Manhattan distance is used, then centroids are computed as the component-wise median rather than mean [15].

#### Expectation Maximization (EM)

The EM algorithm [12] creates partitions assigning a probability distribution to each instance. EM can decide how many clusters to create by cross validation, or is possible to specify apriori how many clusters to generate [15].

#### Adaptive Quality-based Clustering Algorithm (AQBC)

It's a heuristic iterative two-step algorithm with computational complexity approximately linear. The first step consists in finding a sphere in the high-dimensional representation of the data where the density of expression profiles is locally maximal. In a second step, an optimal radius of the cluster is calculated based only on the significantly coexpressed items which are included in the cluster. By inferring the radius from the data itself, there is no need to find manually an optimal value for this radius by trial-and-error [13].

#### K-Medoids

It's an exact algorithm based on a binary linear programming formulation of the optimization problem [21], using ‘lp’ from package ‘lpSolve’ as solver [16]. Probably is not possible to obtain clustering solutions depending on available hardware resources due to the quadratic order of the program. The K-Medoids R implementation is an NP-hard optimization problem. Partitioning Around Medoids (PAM) [14] is a very popular heuristic for obtaining optimal K-Medoids partitions [16].

#### MakeDensityBasedClusterer (MDBC)

It’s an algorithm wrapping the SimpleKMeans and possibly others clusterers algorithms. Makes SimpleKmeans return a distribution and density. Fits normal distributions and discrete distributions within each cluster produced by the wrapped clusterer. For the SimpleKMeans supports the number of clusters requestable [15].

### Cladograms

The clustering operations were made by calculating the Euclidean distance from the first alphabetically ordered species, defined as the pivotal species, to all the other species. Therefore, when ASAP created the clusters, it already had a symmetric distance matrix containing a data set with all the species. All we needed to do was to create a phylogenetic tree expressed as a Newick phylogenetic tree. We developed an unrooted tree created by the software NEIGHBOR from the PHYLIP package. We drew the unrooted tree in Figure 7, representing the eight clusters of the 60 species from dataset2. All default parameters were used.

## Competing interests

The authors declare that they have no competing interests.

## Authors' contributions

MAS encouraged the research and writing, BMC application, provided references and applied mathematical knowledge and gave final approval of the version to be published. ARS downloaded all the data and conducted all the tests, decided to use Linnaean taxonomy as a measure of cluster quality, developed the algorithm and wrote the paper.

JB made substantial contributions to conception and design, analysis and interpretation of data. VAA encouraged submission to BMC and gave final approval of the version to be published. JAM, GCO, AM and AS have given final approval of the version to be published.

## Supplementary Material

Additional file 1 **Qualitative cluster measures.** In this document, we elaborate on aspects of the qualitative cluster measures that are not discussed in this paper, such as the demand for specific metrics for clusters based on Linnaean taxonomic classification, how sequences size influence kdcSearch, a proof that amino acid trigams do not occur by chance, how to make a graphic cluster approximation by cladograms, how the evaluated algorithms were executed and the kdcSearch algorithm pseudo-code. Click here for file

Additional file 2 **Scilab algorithms and raw data.** In this file, we elaborate on aspects of the algorithms and data used in this research. Algorithms were written in Scilab version "5.2.0.1266391513", scilab-5.2.1. Click here for file
