# Supplementary material for: A singular value decomposition approach for improved taxonomic classification of biological sequences
Source: BMC Genomics. 2011 Dec 22;12(Suppl 4):S11. doi: 10.1186/1471-2164-12-S4-S11 (PMC3287580; doi:10.1186/1471-2164-12-S4-S11)
Supplement: Additional file 1 — Qualitative cluster measures. In this document, we elaborate on aspects of the qualitative cluster measures that are not discussed in this paper, such as the demand for specific metrics for clusters based on Linnaean taxonomic classification, how sequences size influence kdcSearch, a proof that amino acid trigams do not occur by chance, how to make a graphic cluster approximation by cladograms, how the evaluated algorithms were executed and the kdcSearch algorithm pseudo-code. [file 1471-2164-12-S4-S11-S1.pdf]

Supplementary material for “A singular value decomposition approach for improved taxonomy classification of biological sequences”

Anderson R. Santos[1]*, Marcos A. Santos[2]*, Jan Baumbach[3], John A McCulloch[1], Guilherme C. Oliveira[4], Artur Silva[5], Anderson Miyoshi[1], Vasco Azevedo[1§]

[1] Department of General Biology, Instituto de Ciências Biológicas, Universidade Federal de Minas Gerais, Belo Horizonte, Av. Antônio Carlos, 6627, MG, 31.270-901, Brazil

[2] Computer Science Departament, Instituto de Ciências Exatas, Universidade Federal de Minas Gerais, Belo Horizonte, Av. Antonio Carlos, 6627, 31.270-901, MG, Brazil

[3] Max Planck Institute for Informatics, Campus E2 1, Saarbrücken, Germany

[4] CEBio and Laboratory of Cellular and Molecular Parasitology, Instituto René Rachou, Oswaldo Cruz Foundation, Belo Horizonte, Av. Augusto de Lima 1715, 30190-002, MG, Brazil

[5] Genome and Proteome Network of the State of Pará, Universidade Federal do Pará, Belém, R. Augusto Corrêa, 66.075-110, PA, Brazil

*These authors contributed equally to this work

[§]Corresponding author

Email addresses:

ARS: asantos.icb.ufmg.br@gmail.com

MAS: marcos@dcc.ufmg.br

JB: jbaumbac@mpi-inf.mpg.de

JAM: johnmcc@usp.br

GCO: oliveira@cpqrr.fiocruz.br

AS: asilva@ufpa.br

AM: miyoshi@icb.ufmg.br

VA: vasco@icb.ufmg.br

In this document, we elaborate on aspects of qualitative cluster measures that are not discussed in the paper, such as the demand for specific metrics for clusters based on Linnaean taxonomic classification.

Summary

kdcSearch algorithm pseudo-code 4

Clusters Quality Measure....................10

General clusters quality measure....................12

Sequence size matters....................13

Amino acid trigams do not occur by chance....................17

Graphic cluster approximation by cladograms....................19

Clustering algorithms evaluated....................19

R: statistical software....................19

Weka: Data Mining Software in Java....................20

K-Means-R....................20

K-Means-WEKA....................20

Expectation Maximization (EM) 20

Adaptive Quality-based Clustering Algorithm (AQBC) 21

K-Medoids....................21

MakeDensityBasedClusterer (MDBC)....................22

Figures....................23

Figure S1 - Cluster box plot obtained from Table 5 (BMC)....................23

Figure S2 - Trigrams probability distribution of the dataset2....................24

Figure S3 - Trigrams probability distribution of the complete proteome of a bacterium..25
Tables..26
Table S1 - Species randomly selected to compose the progressive shortening sequence size for the sequence size impact test..26
Table S2 - Sequences size impact within ASAP clusters..27
Table S3 - Example of 3-gram frequency matrix..27

kdcSearch algorithm pseudo-code

The algorithm kdcSearch is basically composed of two procedures that intercalate during execution. The first procedure explores the possibilities of formation of partitionings of species according to different values of kdc. The second procedure analyzes the quality of these partitionings based on Linnaean taxonomy. These procedures begin with this main code:

```
CREATE FUNCTION AS main()
VAR n = 76; --How many species in the initial dataset
VAR EDRD = round( n/3 ); --Determines an Empirical Dimensional Range Division
--NULL means 'no targeted species' so process all of them
VAR M = ExplorePossibilities( NULL, EDRD );
-- rank k=n species means no SVD
VAR DistanceMatrix = create_distance_matrix_by_SVD_rank_reduction ( M, n );
kdcSearch ( DistanceMatrix, EDRD );
END.
```

```
CREATE FUNCTION AS ExplorePossibilities ( TargetedSpecies, EDRD )
-- Determines all possible partitions/clusters amounts according to rank k and distance d
-- Parameter 1: TargetedSpecies = Species ID for work on
-- Parameter 2: EDRD = Determines an Empirical Dimensional Range Division. It must be lesser than or equal n
BEGIN
VAR k; --SVD rank value
VAR d; --Euclidean distance
VAR c; --dataset partitions/clusters amount
--dataset: 76 mitochondrial amino acid sequences including a positive control group with 10 species and a  negative control group with 12 species
VAR dataset = read_fasta_from_file('dataset2.fasta'); --Read amino acid fasta file
if (TargetedSpecies is not NULL){
    dataset = Remove_Untargeted_Species_FromDataset(dataset, TargetedSpecies);
}
VAR n = size( dataset ); --count species amount
VAR M = create_amino_acid_3gram_frequency_matrix( dataset );--create a similarity distance matrix from data
VAR kdelta = round( n/EDRD ); -- Determines rank increment according to EDRD
```

```
for( k = kdelta; k <= n; k = (k + kdelta) ){ --Explore multiple rank dimensions ...

    VAR DistanceMatrix = create_distance_matrix_by_SVD_rank_reduction ( M, k ); --Create M aproximation matrix with rank k

    VAR X = max( DistanceMatrix ); --Determines the maximum distance among all species

    VAR Y = min( DistanceMatrix ); --Determines the minimum distance among all species

    VAR EEDD = 50; --Determines an Empirical Euclidean Distance Decrement to create different data partitions

    for( d = X; d >= Y; d = (d - ( d/EEDD ) ) ){ --Explore multiple Euclidean distances with small incremental values

        c = create_partitions( DistanceMatrix, d ); --Calcule c=f(d)

        write_line_to_file('dataset2.clusters.result', k, d, c); --Store data for further evaluations

    } --end for d

} --end for k

return M;

END;
```

```
CREATE FUNCTION AS kdcSearch ( pM, EDRD ) --Receives similarity distance matrix from data as parameter

--Create partitions between a range of k values and search for kdc values maximizing cLtlf

--Parameter 1: Symmetric distance matrix derived from amino acid 3-gram frequencies

--Parameter 2: EDRD = Determines an Empirical Dimensional Range Division. It must be lesser than or equal n

        --Suppose one decides to explore cluster quality ranging from 5 to 30 clusters, with increments of 5 clusters.

        --For this purpose, first of all, one needs to gather the first Euclidean distance that yields such partitions between all rank values.

VAR k; --SVD rank value

VAR d; --Euclidean distance

VAR c; --dataset partitions/clusters amount

VAR n = size( pM ); --count species amount

VAR kdelta = round( n/EDRD ); -- Determines rank increment according to EDRD

VAR cLtlf    { value=0; k; d; c; DistanceMatrix }; --creates a record to store five variables: cLtlf, k, d, c and DistanceMatrix

VAR cLtlf_max{ value=0; k; d; c; DistanceMatrix }; --creates a record to store the best five variables

VAR clusters_result = read_matrix_from_file('dataset2.clusters.result'); --read stored data about partitioning possibilities

VAR KeepGoingDown = TRUE;
```

```
for( k = kdelta; k <= n; k = (k + kdelta) ){ --Walk through multiple rank values

        for( c = 5; c <= 30; c = c + 5){ --Walk through multiple partition sizes

                d = find_first( k, c);     --find first distance yielding c partitions

                VAR DistanceMatrix = create_distance_matrix_by_SVD_rank_reduction ( pM, k ); --Create M aproximation matrix with rank k

                cLtlf = compute_cLtlf( k, d, c, DistanceMatrix ); --Computes de common Linnaean taxonomic level frequency (cLtlf)

                if cLtlf_max.value < cLtlf.value { --If found a new max cLtlf then make it the new max record ...

                        cLtlf_max = cLtlf; --... containing rank k and number of partitions c=f(d) for biological significance.

                } --if cLtlf_max

                Plot_Graphics (k, d, c); --This data plus taxonomic  levels were used to create Figures 5, 6

                --This is a critical point: if it was possible to isolate the positive control group in a single partition ...

                --... the algorithm no longer needs to perform a recursive call for the better scored partition ...

                --... according Linnaean taxonomy. Here we only need to keep in mind that this algorithm run is enough to ...

                --... create clusters with biological meaning.

                if ( Positives_Isolated( cLtlf ) )--if positives were isolated in a single partition then stop recurrences.

                        KeepGoingDown = FALSE;

                }

        } -- end for c
```

```
} -- end for k
print (  'Stronger biological significance=' cLtlf_max.value ' with rank=' cLtlf_max.k
                    'and distance=' cLtlf_max.d ' creating ' cLtlf_max.c ' clusters' );
         --Taking into account Figure 5 this print shall shows: cLtlf_max.value=480, cLtlf_max.k=39, cLtlf_max.d=68.88, cLtlf_max.c=15
          if ( KeepGoingDown == TRUE){ --if positives were not isolated in a single partition then keep going with recurrences.
          --Now, the search continues with the better scored partition. For this purpose, remove other partitions without positives
          DistanceMatrix = isolate_species_partition_with_max_cLtlf( cLtlf_max ); -- Concentrate on the partition with positives
          VAR TargetedSpecies = determines_new_focus_species( DistanceMatrix ); --Create a list of remaining species names
          EDRD = ( size(TargetedSpecies)/3 ); --Determines an Empirical Dimensional Range Division.
          ExplorePossibilities ( TargetedSpecies, EDRD ); --Create new values k, d, c for the reduced data set
          kdcSearch( DistanceMatrix, EDRD ); --Recursive call, but only with species not so distant from positives.
}else{--if not KeepGoingDown so finalize the run
          Finalize ();
}--if KeepGoingDown
END;
```

Clusters Quality Measure

Linnaean taxonomy was used to rate each cluster created by our algorithm. We searched all taxonomy levels and respective values for each of the 76 species. For example, *Aythya americana* (Aame) belongs to the family (taxonomy level) *Anatidae* (taxonomy level value) and to the genus *Aythya*. It is difficult to obtain all possible Linnaean taxonomy levels for a large data set, because not all species have official classifications at all possible levels. Consequently, we filtered all common taxonomy levels from the original 64 species; 14 levels were used to rate the clusters. These levels are listed in decreasing hierarchical order: Superregnum, Regnum, Subregnum, Cladus1, Phylum, Subphylum, Infraphylum, Classis, Subclassis, Ordo, Familia, Genus and Species. However the Species level was not included, because it is the level that we sought to prove to be related, consequently, 13 levels were used.

It was necessary to include these 12 species in the data set, because otherwise all possible clusters from the original 64 species would have at least eight equal common taxonomy level values, among the 13 levels that we tested, due to the relatively close relatedness among them, with more than 60% of all levels in common. Some of the additional 12 species incorporated into this data set did not have all 13 levels in common, because they are relatively unrelated, considering Linnaean taxonomy, so that even the number of necessary classification levels to distinguish those species differs. This becomes clear when one considers a large set of vertebrate species in the same data set, together with an arachnid, a sea anemone, a fungus, a marine green alga, a jellyfish, a coral and a starfish. Consequently, when information concerning one or more of these 13 taxonomy levels was lacking, our solution was to repeat the previous non-void level, so that our algorithm did not compare a non-void level name with a void level name, when measuring cluster quality.

To measure the quality of clusters across different SVDs, based on increasing rank value, it was necessary to make sure that the number of generated clusters was kept constant while using ASAP. To achieve such a constant number of clusters across different rank values, it is necessary to previously determine which distance parameters, the d values, increase such cluster numbers. For example, if the d value is too high, all species are clustered into just one cluster; on the other hand, if the d value is too small, each species is clustered into its own individual cluster, resulting in this case in 76 different clusters. None of these hypothetical situations are desirable in our system, because we want some degree of separation that allows us to make considerations about the relatedness of species. In order to establish a d value for each rank value, we ran the ASAP algorithm, producing all possible clusters as a function of a small increase in the d value, from rank value 3 to 63, adding steps of four ranks in the 64 species data set. We also ran the ASAP algorithm between rank values 3 and 75, with step value 3, to obtain a d value that maintains the number of clusters within the 76 species data set constant. In this particular case, we wanted to know how cluster quality is influenced by rank value, so the number of clusters was varied from 5 to 30, with a five-cluster step between each algorithm run.

A relevant question that one could make is: “Why didn’t the 76 species data set give good clusters, given that it includes more elements and more diversity than the 64 species data set?” The answer is simple. Along with more elements and more diversity, the 76 species data set also includes such diverse species that our algorithm never joins groups of them together into the same cluster unless the number of clusters is too small. For example, when we cluster the 76 species data set, the Aves classes are correctly clustered when the desired number of clusters is 15 or higher. In this case, most clusters consist of a single species. On the other hand, when the 64 species data set is clustered,

the Aves classes are correctly clustered when the desired number of clusters is five. So, by choosing the 64 species data set, we avoid dealing with a large number of clusters containing a single element and no additional information.

Since the used ASAP algorithm does not keep track of changes in cluster positions across rank value variations, when clusters have few elements (less than four elements), it could be difficult to perceive differences when elements are added or excluded. We focused on clusters with more elements, in order to easily obtain evidence to conclude that the cluster observed with rank ki+3 or ki+4 is the same cluster obtained with rank ki, even after some elements have been added or excluded. These elements changes are responsible for increases or decreases in quality, as a function of the number of common Linnaean taxonomy levels.

General clusters quality measure

The differentiation of clusters measured by the number of Linnaean levels in common among species was effective in identifying the appropriate amount of clusters and singular values to be used for the separation of species into groups with biological significance. However, when examining the average quality of clusters obtained by cLtlf, we are unable to discern the quality of the separation of species (Figure S1); s another measure of quality that allows inferences about the overall effectiveness of clustering is necessary. In this work, we used a measure of quality to infer how species were separated into different clusters, measuring the overall effectiveness of the separation of all species at each new stage of separation. This quality measure demonstrated which clustering algorithm was more efficient and the number of singular values that would give the most effective clustering.

To measure the quality of a set of clusters we decided to take into account two basic parameters. We found that the quality of the clustering process was directly proportional

to the sum of cLtlf of all clusters. At the same time, we observed that there is a wide variation in the quality of clusters. A large variation in quality of clusters can be synonymous with clusters with few elements, which makes inferences about similarity between species difficult. For example, in creating clusters by using ASAP and the array of trigram frequencies of amino acids with full rank (Table 5, BMC Bioinformatics), four clusters were generated with only one element. Thus the quality of a set of clusters is inversely proportional to the variation in the quality of clusters measured by cLtlf. We decided to use the standard deviation to express the variation in the quality of cLtlf. The deduced formula (1) from these considerations was applied to four sets of clusters; the results are displayed in Table 1, in the column with the heading 'Linnaean clusters quality' (Lcq).

$$Lcq = \frac{\sum cLtlf}{\sigma} \qquad (1)$$

It is clearly shown by the analysis of Lcq in Table 1 that the ASAP algorithm performance was almost two times lower than that of K-Means, both using the non decomposed matrix of trigrams and the distance matrix obtained from the singular value decomposition with estimated optimum parameters. We also observed that the matrix rank reduction produced clusters with better quality than the matrix that was not decomposed into singular values for K-Means and ASAP.

Thus we showed that K-Means was effective in producing clusters with reasonable biological significance using a matrix of trigram frequencies of amino acids obtained by SVD, with the number of clusters and the number of singular values systematically analyzed by the methodology suggested in this work.

Sequence size matters

To assess the impact of amino acid sequence size on the creation of clusters, we systematically decreased the mitochondrial protein sequences size for some species. The

18 species in Table 1 were used to evaluate the impact of sequence size on ASAP algorithm results. The choice of the species was random, but the groups they belonged to were not randomly chosen. Selected groups were created in the second recurrence clustering, using nine singular values and 60 species. These groups are shown in Table 6 (BMC Bioinformatics). The group in Table 1 that had the fewest species had four species. All species of each group had at least eight Linnaean levels in common. The criterion chosen was to select at least half of the elements of each of these groups to study how they would be separated when the sequence sizes were reduced. In this random sample, the largest sequence size differed from the smallest by only 26 amino acids; the largest was composed of 3,835 amino acids.

Tests were made considering decreasing sizes from 100 to 50%, with intervals of 5% in the amino acid sequence size. The objective was to define the maximum percentage difference between sequences of amino acids analyzed with clustering algorithms that would cause a misclassification. In this case a misclassification means a classification based only on the size of the amino acid sequences, independent of the amino acid components of the proteins. Groups created with 100% of the amino acid sequences were considered the initially correct data set. These groups with 100% of the sequences were compared with groups created with 95% of the sequences of the 18 species; as the amino acid sequences varied among tests, we called them mutable sizes. The remaining 36 species were regarded as immutable, a group that always maintained the full size of the sequences through the tests. This is important to determine when the parameters that shape the singular value decomposition start becoming ineffective for separating species of the sequences when sizes vary widely. The parameters number of singular values used by SVD and the number of clusters were not changed, maintaining the same parameters that generated groups with 100% of the size of the sequences. The only

variable between the test results was size of the amino acid sequences.

Table 2 exhibits the comparative results between different tests. This table does not show which species changed groups; it indicates the number of species that were no longer in their original group. The group is indicated in the leftmost column. Here, we did not record when species changed from partitions, since this data was not useful for this analysis, except when a species clustered with immutable elements and switched to a group that did not have any immutable element. In the various tests, a mutable element rarely exchanged groups, and when it did, it moved to a group that also had immutable elements. When a mutable element changes to a group that has no immutable elements, we assume that the parameters used by SVD and number of clusters begin to lose the ability to group species according to homology; this event is penalized twice. If each group exchange has a fee of -1 then the penalty for changing to a group that does not have an immutable element is -2 (-1 * 2). The first reference (100 versus 95%) with only one species changing to another group was classified, but the target group had other immutable elements, resulting in a penalty of only -1. In the second round, we compared groups having the 18 species with 95% size versus the groups possessing these same species with 90% size. Now the group that was considered correct contained mutable species with 95% of the size of their amino acid sequences.

Two species were classified into other groups compared with the previous groups, but these two species formed a group with no other immutable or mutable species. The species 'Lcha' and 'Porn' were together in previous rounds and now got back together, but in a separate group. Using the rule for penalties, this group should have a double penalty -4 (-2 * 2). In the third round, we compared the group with the 18 species with 90% of the original sequence size, now considered the correct one, versus the groups possessing these same species with 85% of the sequence size. And so on, until we

compared groups where 18 species were mutable going from 55 to 50% of sequences size. The inability of the algorithm to discern species in the second round is questionable, because even so a separate group was created with two species that were together in the previous rounds. Still following this reasoning, after the third round (90 versus 85%), we could not correctly classify five of 18 species. The evidence for this lack of discernment between species is that these five species left groups that had immutable species and began to compose a unique group of species with only mutable elements, generating a double penalty. In the fourth round (85 versus 80%), 10 other species came from groups that also had species with immutable sizes and joined the group created in the third round (90 versus 85%), which only had species with mutable sizes. In the last round (55 versus 50%), all the 18 mutable size species were isolated in a single group, without species with immutable size sequences.

Consequently, we can observe that differences between the sizes of the amino acid sequences can be tolerated up to 5% by ASAP, without having to recalculate the parameters that achieve adequate separation of species. Using 5% sequence size differences, we are taking a huge risk that amino acid sequences will be grouped only by the smallest trigram frequencies, no longer considering differences in the frequency of trigrams that are meaningful to distinguish between evolutionary closely-related species.

Each loss has -1 penalty. When a group loses an element that goes to another group only with mutable size elements, then the penalty is doubled. The creation of groups with mutable size elements is evidence of inadequacy in the reuse of the parameters ‘number of singular values’ used by SVD and ‘number of clusters’. We emphasize that the parameters that create the matrix of distances between species may lose their ability to discriminate between sequences of related species. In fact, all these rounds were made

with a distance matrix decomposed into a fixed number of singular values and ASAP always creating a fixed number of clusters, independent of specific characteristics that each data set possessed. To ensure good quality of clustering between multiple data sets, we can calculate the correct rank value again and see what value always properly separates the control group (in this case the *Aves* group). So, even though the size of the sequences decreases, it is still possible to properly separate the species using smaller portions of their amino acid sequences. To avoid having to repeat the kdc parameters finding procedure, whenever a new element is included in the data set, one option would be to evaluate whether the size of this new element is within the margin of safety that enables the algorithm without changing parameters. For cases in which many species start to appear in a single group, the solution is to run a new parameters finding round on this particular group.

Amino acid trigams do not occur by chance

We used trigrams (N-grams, with N = 3) amino acids to establish homology between the sequences of species and classify them into clusters. Considering the 20 most common amino acids, each trigram that is observed is mapped into one of 8,000 (20^3) possible combinations of trigrams . An example of this representation can be seen in Table 3. If we add all columns of each row, we obtain the frequency of occurrence of each of the 8,000 possible trigrams for this particular data set. Dividing these frequencies by the sum of the frequencies of occurrence of all trigrams gives us the probability distribution. Figure S2 shows the probability distribution of all trigrams from dataset2, sorted in descending order. In this relatively small data set, we found 7,248 trigrams. The 25% most frequent trigrams accounted for 76% of all occurrence probabilities in the entire data set, while the 25% least frequent accounted for only 1.3% of the probability. These percentages clearly show that the distribution of amino acid

trigrams is not random. In dataset2 each species is represented by 13 sequences of mitochondrial proteins that are known to be highly conserved throughout evolution. Taking into account dataset1, we find the maximum and minimum sizes of sequences, 3,861 (Dvir) and 3,771 (Scam). The size difference is only 91 amino acids. Since the sizes of the amino acid sequences are so close, and there is a high degree of similarity among them (67%), it could be argued that the method of this algorithm to classify species by amino acid trigrams only has the ability to distinguish species when these two conditions are met (close sequence sizes and high similarity); otherwise there is a chance of having trigrams of amino acids distributed randomly. A counter argument can be used against this randomness hypothesis. It is widely known that biological sequences can have more frequent amino acid or nucleotides sets, which is a supporting pillar for classic algorithms that align sequences. If the hypothesis of randomness takes into account similarity level and sequence size, for example, the trigram distribution of amino acids should be random when trigram frequencies for the entire proteome of an organism are computed. The complete proteome of an organism has proteins with greatly varied sizes and functions that are just as varied. To try to answer this question we analyzed the whole genome trigram probability distribution of *Corynebacterium pseudotuberculosis* strain 1002.

In this data set, we found 7,987 trigrams, almost the whole range. The 25% most frequent trigrams accounted for 63% of all occurrence probabilities in the whole proteome. The 25% least common accounted for only 3.5%. This result does not support the hypothesis of randomness, as seen in Figure S3, which shows a probability distribution similar to the probability distribution of Figure S2. This feature provides evidence to conclude that when we use trigrams of amino acids to analyze biological sequences we find non-random distributions (flow distribution), allowing the use of

trigram frequencies to distinguish different proteins among species and even within the same species, as we demonstrated in analysis of the proteome of *Corynebacterium pseudotuberculosis* strain 1002.

Graphic cluster approximation by cladograms

Our purpose was to develop a graphic view of the clustered elements that could provide a more comprehensible picture than just a set of alphanumeric information about the data. We identified the clustered elements with graphic labels, instead of alphanumeric information. In Figure 7 (BMC), clusters obtained and described in Table 3 are labeled in blue (Clusters 1 to 8). When the cluster label is near a stem, this means that following the stem to the species labels (attached to the ends of branches), all species within the branch belong to the cluster denoted by the blue label. If, however, the cluster label is not shown next to a stem, but rather next to the species labels, this means that the species belonging to that particular cluster do not pertain to the cluster indicated next to the stem upstream. It is evident that this cladogram does not exactly represent the clusters created by the clusterization technique presented here. However, it is also evident that though it is not an exact representation of the clusters, it is a very similar approximation, with few species represented in groups different from what is indicated by the clusters. Therefore, we understand that this phylogenetic tree represents a very good approximation of Linnaean relationships, revealed by clusters that have high biological value based on comparison with taxonomic information. This representation could be used as an alternative to visualizing these clusters as graphs.

Clustering algorithms evaluated

R: statistical software

The software GNU R statistical computation and graphics system was installed using the operational system Linux, distributed by Ubuntu, version 10.10, through the command '*sudo apt-get install r-base*'.

Weka: Data Mining Software in Java
The software Weka was downloaded from http://www.cs.waikato.ac.nz/ml/weka/, installed and executed according to instructions on this site. This software executes the clusterization algorithms EM, SimpleKMeans (K-Means-WEKA), and MakeDensityBasedClusterer.

K-Means-R
We used the K-Means algorithm available in the normal installation of the statistics software R. The distance matrices between species, constructed with k=60 and k=9, were loaded into variables of the software R, without column headings and with a mnemonic identifier for each species in column 1. The *kmeans* algorithm was run with the following commands: *kmeans(K60[,2:61], 8, nstart = 30)* e *kmeans(K09[,2:61], 8, nstart = 30)*. The list of results was manually associated with the names of the species for evaluation of the results.

K-Means-WEKA
The distance matrices with k=60 and k=9 in CSV format had a heading in the first line, as well as in the first column, with mnemonics for each species. The only parameter that was modified among the standard parameters presented by the software was the number of clusters (-N 8) equal to eight. The Linux command line that executed the algorithm was: *java -cp weka.jar weka.clusterers.SimpleKMeans -t stuart.60.fasta.md.K09.weka.csv -N 8 -A "weka.core.EuclideanDistance -R first-last" -I 100 -S 10.* This –t specific command specifies the file with the distance matrices, -N the number of clusters desired, -A the type of distance involved, -I the maximum number of interactions, and -S is the randomization seed.

Expectation Maximization (EM)
The same preparation of K-Means-WEKA data was used because it involved the same processing environment, the software WEKA. The only parameter that was modified among the standard parameters presented by the software was the number of clusters (-

N 8) set at eight. The Linux line command that executed the algorithm was: *java -cp weka.jar weka.clusterers.EM -t stuart.60.fasta.md.K09.weka.csv -I 100 -N 8 -M 1.0E-6 -S 100.* In this command -t specifies the file with the distance matrix, -I the maximum number of interactions, -N the desired number of clusters, -M the minimum standard deviation allowed and -S is the randomization seed.

Adaptive Quality-based Clustering Algorithm (AQBC)

The software Java Machine Learning Library 0.1.5 library was obtained from http://java-ml.sourceforge.net/, installed and run according to instructions available from the site. A small java class was developed and compiled to use the software package "net.sf.javaml.clustering.AQBC", which processed the same distance matrices used by K-Means-R. Executing this code gave this command line: A *java myAQBC stuart.60.fasta.md.K09.javaml 0 0.20 true*. The first parameter is the name of the distance matrix file, the second indicates that the species mnemonics are in the first column (0), the third is the desired level of significance and the fourth is an option to normalize the data. The latter two parameters were selected so that eight clusters would be produced, so that comparisons could be made with the other algorithms that we tested in this study.

K-Medoids

The algorithm kmedoids is not standard in the installation of the software R. In order to install it, it was necessary to execute the following command with administrative privileges:

install.packages("clue", dependencies = TRUE);

The same preparation of K-Means-R data was used because the same processing environment was involved. The algorithm kmedoids was run with the following commands:

K60=read.table("stuart.60.fasta.md.K09.javaml", header=FALSE);

library(clue);

kmedoids(K60[,2:61], 8);

The first command read the distance matrix, the second loaded the library with the algorithm kmedoids and the third executed the algorithm using as parameters the distance matrix read with the specification of producing exactly eight partitions with the data.

MakeDensityBasedClusterer (MDBC)

The same preparation of K-Means-WEKA data was used because the same processing environment was involved, the software WEKA. The only parameter modified among the standard parameters presented by the software was the number of clusters (-N 8) equal to eight. The Linux line command that executed the algorithm was:

java -cp weka.jar weka.clusterers.MakeDensityBasedClusterer -t stuart.60.fasta.md.K09.weka.csv -M 1.0E-6 -W weka.clusterers.SimpleKMeans -- -N 8 -A "weka.core.EuclideanDistance -R first-last" -I 500 -S 10

In this command -t specifies the distance matrix file, -M the minimum standard deviation permitted, -W the cluster to wrap, -N the desired number of clusters, -A the distance measurement, -I the maximum number of iterations and -S is the randomization seed.

Figures

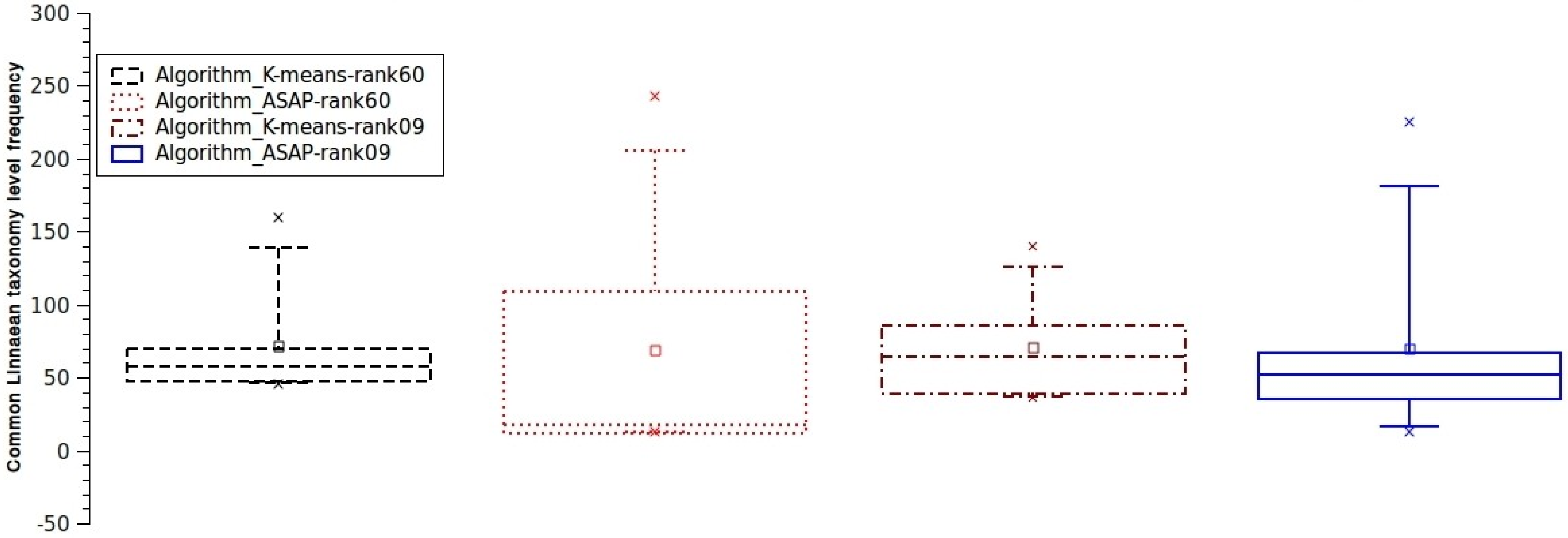

Figure S1 - Cluster box plot obtained from Table 5 (BMC)

There is no significant variation between the clusters around the mean measure cLtlf. The distribution of species in the randomly generated clusters has a cLtlf equal to the average of these four methods. This situation is explained by the small margin of only four Linnaean levels that separate all clusters generated by different algorithms and configuration parameters of these algorithms. It would be necessary to use another measure to infer about the quality of clustering algorithms and their parameters; this metric appears in Table 1.

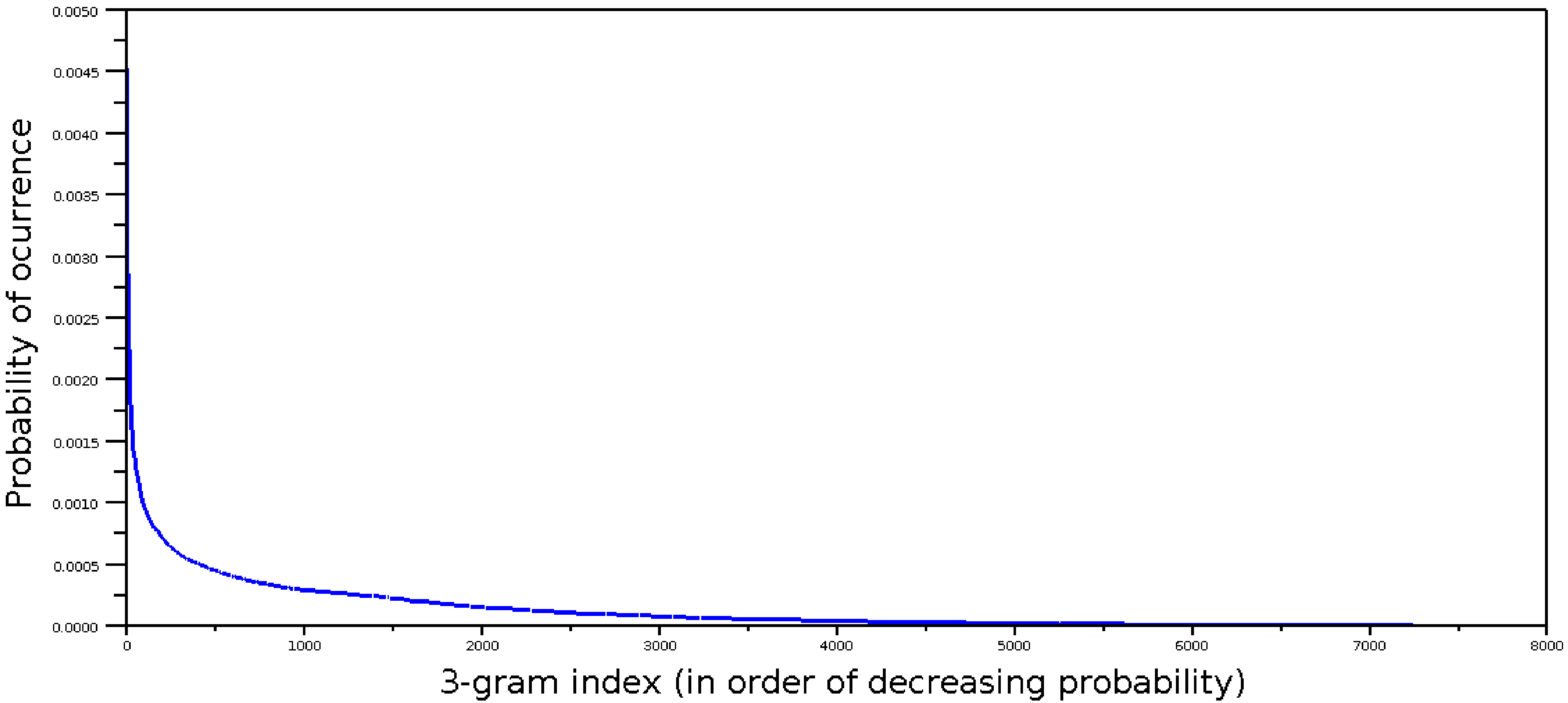

Figure S2 - Trigrams probability distribution of the dataset2

The probability distribution is not random in dataset2.

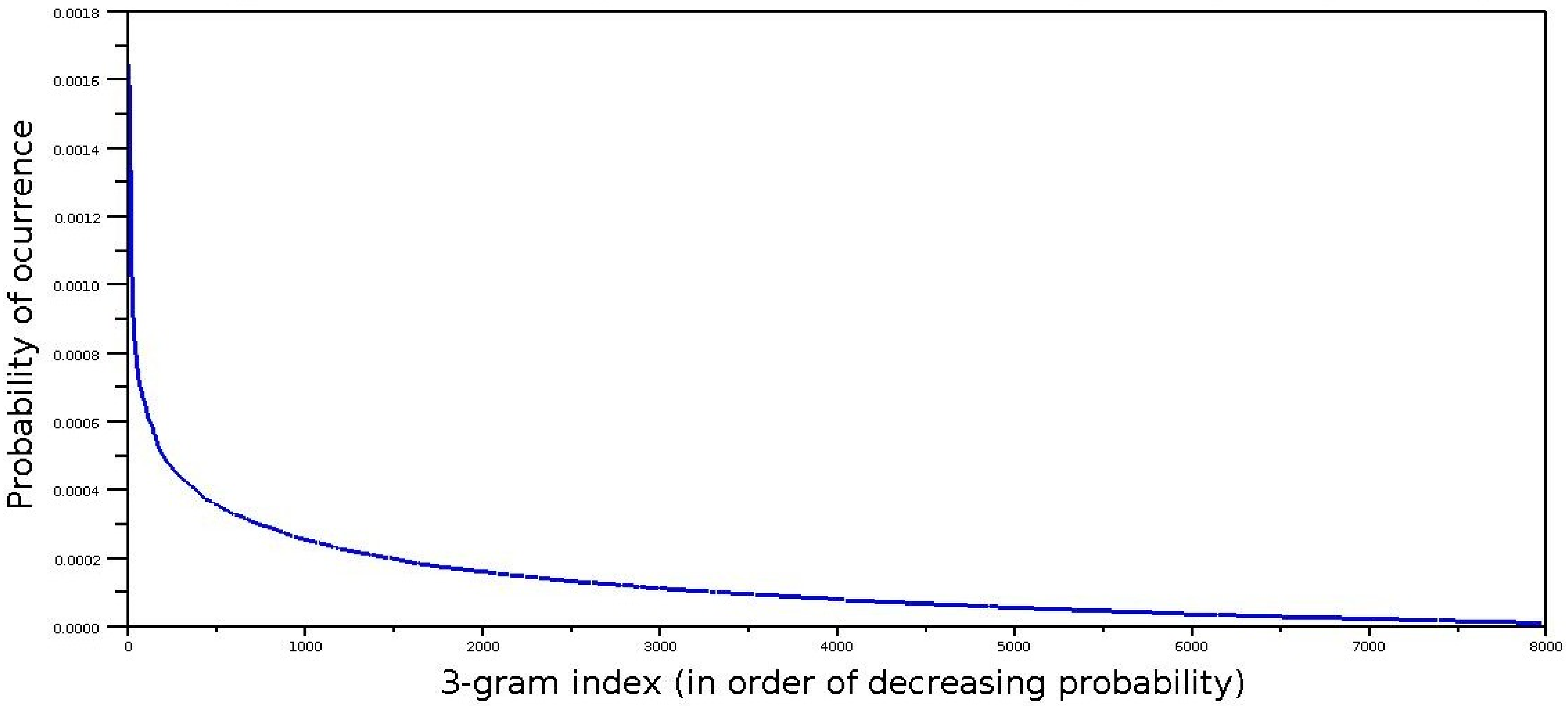

Figure S3 - Trigrams probability distribution of the complete proteome of a bacterium

Probability distribution of 3-gram in the complete proteome of the bacterium *C. pseudotuberculosis* strain 1002. Though they have different sizes and functions, the probability distribution of the trigrams is not random.

Tables

Table S1 - Species randomly selected to compose the progressive shortening sequence size for the sequence size impact test

Species randomly chosen to compose the set that had the amino acid sequences gradually decreased by 5% between rounds. These species formed the group of species with mutable sizes. The results of these rounds were used to determine a size difference between sequences of amino acids that makes correct discrimination between species ineffective.

Species	Original Size	Species	Original Size
Gmor	3,845	Drer	3,829
Sfon	3,839	Teur	3,828
Saca	3,838	Oari	3,828
Poli	3,837	Lcha	3,827
Scan	3,836	Svul	3,826
Omyk	3,833	Oafe	3,825
Pvit	3,832	Porn	3,824
Hgry	3,832	Ocun	3,824
Runi	3,829	Hamp	3,819

Table S2 - Sequences size impact within ASAP clusters

Comparison of results between two clustering rounds taking into account a gradual decrease of 5% in the size of amino acid sequences from 18 species. These 18 species were called mutable size species (Table 7), composing a data set of 54 species. From the second column on, the differences between rounds with mutable size species are shown. The first column shows the number of the group that lost one species to another group. Gain of a species by a group is not represented in this Table.

	**100 - 95**	**95 - 90**	**90 - 85**	**85 - 80**	**80 - 75**	**75 - 70**	**70 - 65**	**65 - 60**	**60 - 55**	**55 - 50**
**1**										
**2**			-3*2	-6*2						
**3**	-1	-1*2								
**4**										
**5**										
**6**									-3*2	
**7**		-1*2				-3*2	-1*2	-2*2		
**8**			-2*2	-2*2						
**9**				-3*2						
**10**				-2*2						
**Sum**	**-1**	**-4**	**-10**	**-26**	**0**	**-6**	**-2**	**-4**	**-6**	**0**

Table S3 - Example of 3-gram frequency matrix

Example of the occurrence of all possible amino acids trigrams (first column) in all sequences of the 76 species (second row).

**3-gram**	**3-gram frequency by species**							
	Aame	Aamu	Aaur	Ajam	Amis	...	Ufoi	Vcha
AAA	8	1	7	11	3	...	9	4
AAC	5	2	13	8	16	...	12	15
AAD	3	19	1	10	0	...	16	11
...	...	...	...	...	...	...	...	...
YYY	2	9	5	7	0	...	5	7
