# Supplementary figures and images for: A singular value decomposition approach for improved taxonomic classification of biological sequences
Source: BMC Genomics. 2011 Dec 22;12(Suppl 4):S11. doi: 10.1186/1471-2164-12-S4-S11 (PMC3287580; doi:10.1186/1471-2164-12-S4-S11)

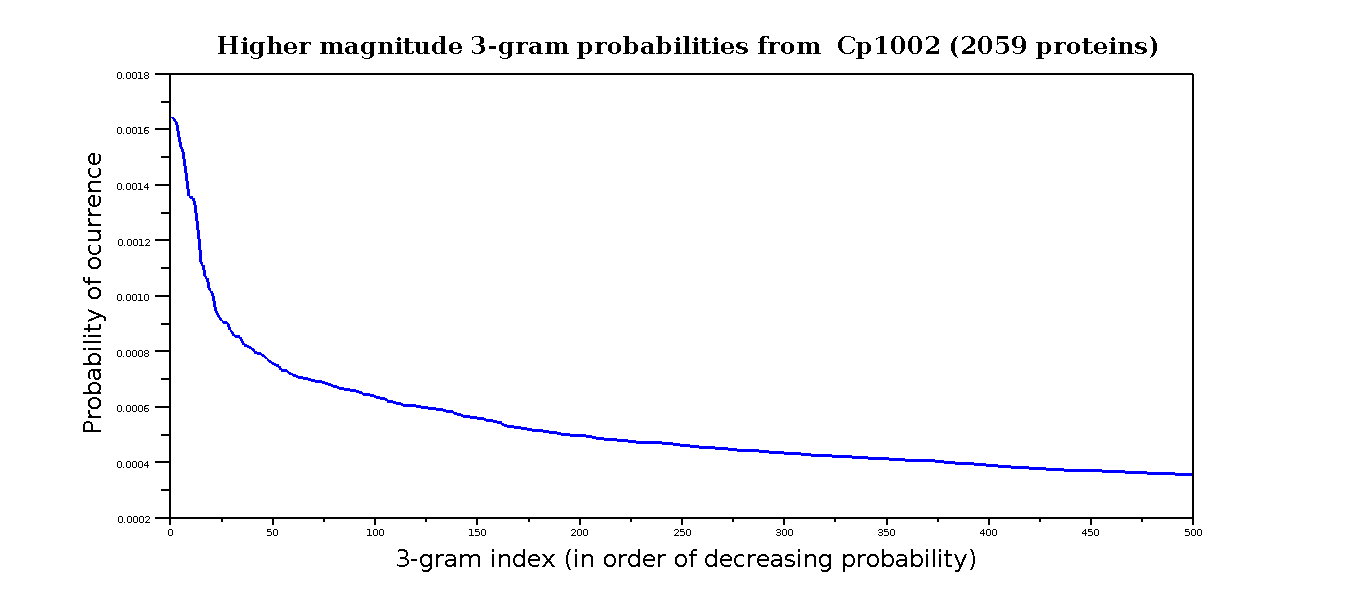

Supplement: Additional file 2 — Scilab algorithms and raw data. In this file, we elaborate on aspects of the algorithms and data used in this research. Algorithms were written in Scilab version "5.2.0.1266391513", scilab-5.2.1. [file 1471-2164-12-S4-S11-S2.zip › SVD_for_improved_taxonomic_classification-S2/Cp/Cp1002_mapgram1_prob_dist.png]

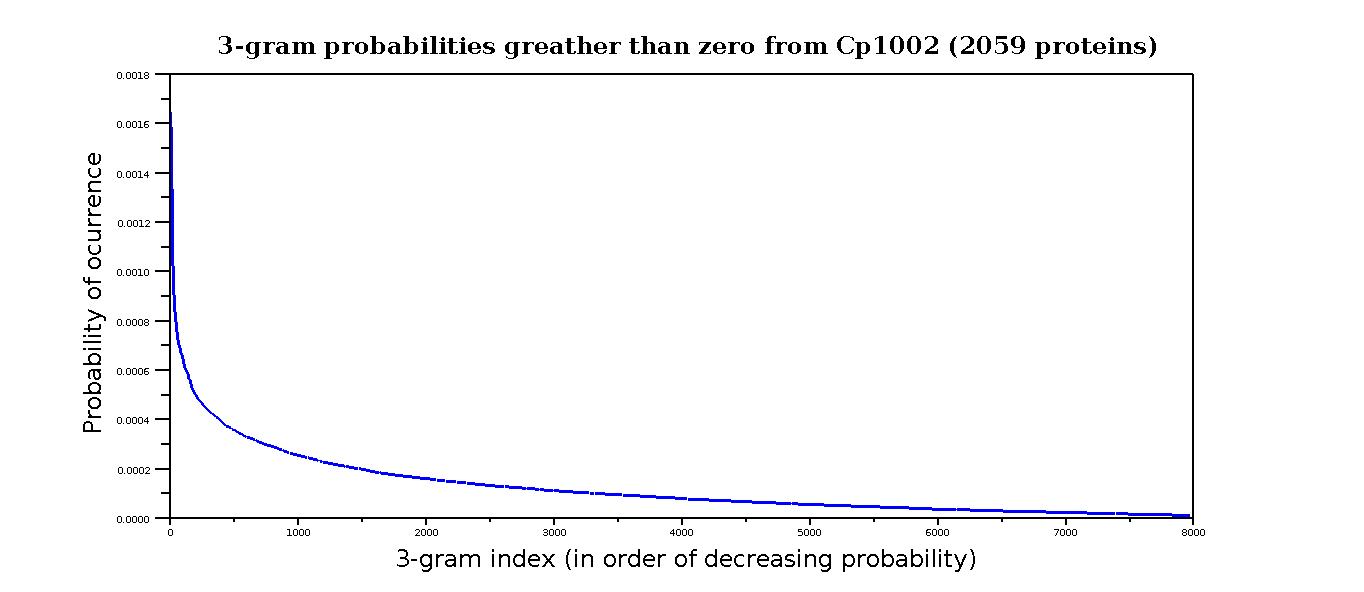

Supplement: Additional file 2 — Scilab algorithms and raw data. In this file, we elaborate on aspects of the algorithms and data used in this research. Algorithms were written in Scilab version "5.2.0.1266391513", scilab-5.2.1. [file 1471-2164-12-S4-S11-S2.zip › SVD_for_improved_taxonomic_classification-S2/Cp/Cp1002_mapgram2_prob_dist.jpg]

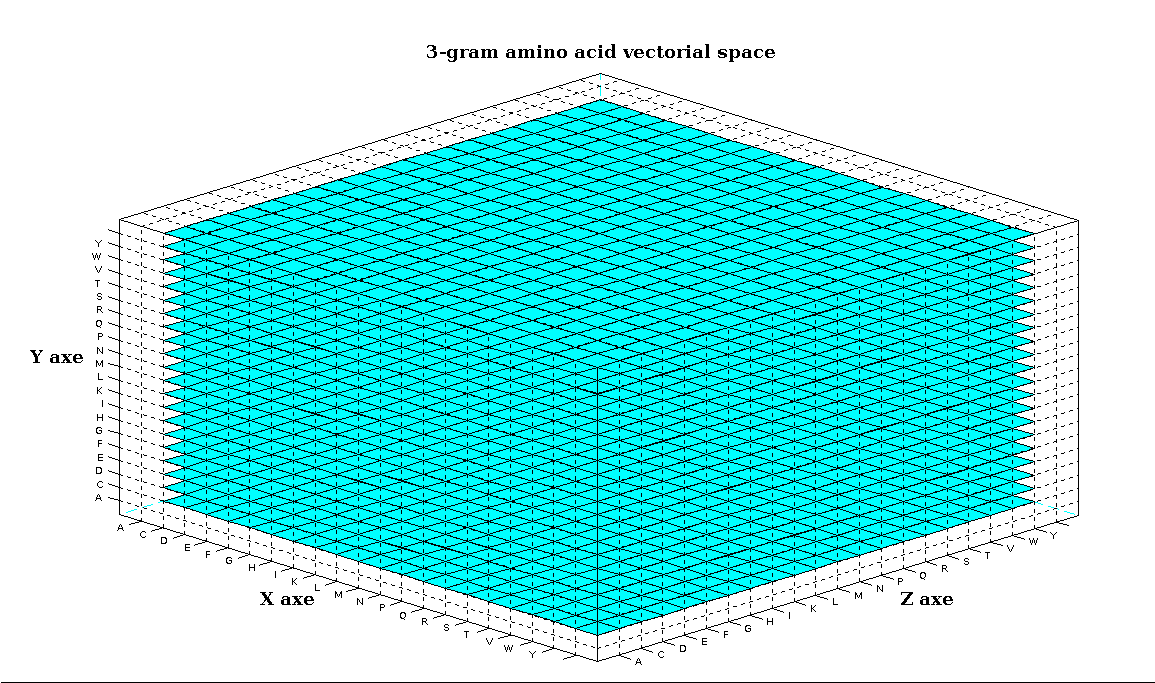

Supplement: Additional file 2 — Scilab algorithms and raw data. In this file, we elaborate on aspects of the algorithms and data used in this research. Algorithms were written in Scilab version "5.2.0.1266391513", scilab-5.2.1. [file 1471-2164-12-S4-S11-S2.zip › SVD_for_improved_taxonomic_classification-S2/76species/3gram/3gram-space.png]

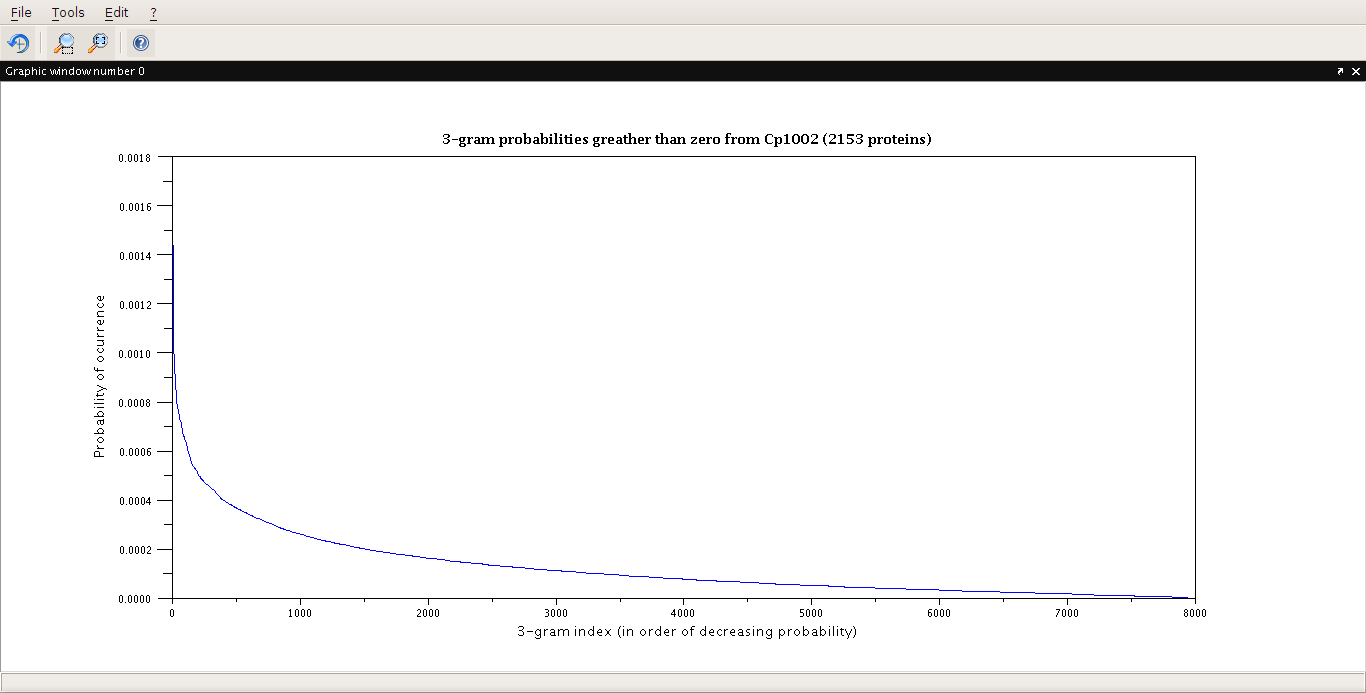

Supplement: Additional file 2 — Scilab algorithms and raw data. In this file, we elaborate on aspects of the algorithms and data used in this research. Algorithms were written in Scilab version "5.2.0.1266391513", scilab-5.2.1. [file 1471-2164-12-S4-S11-S2.zip › SVD_for_improved_taxonomic_classification-S2/76species/3gram/Cp1002-3grams.png]

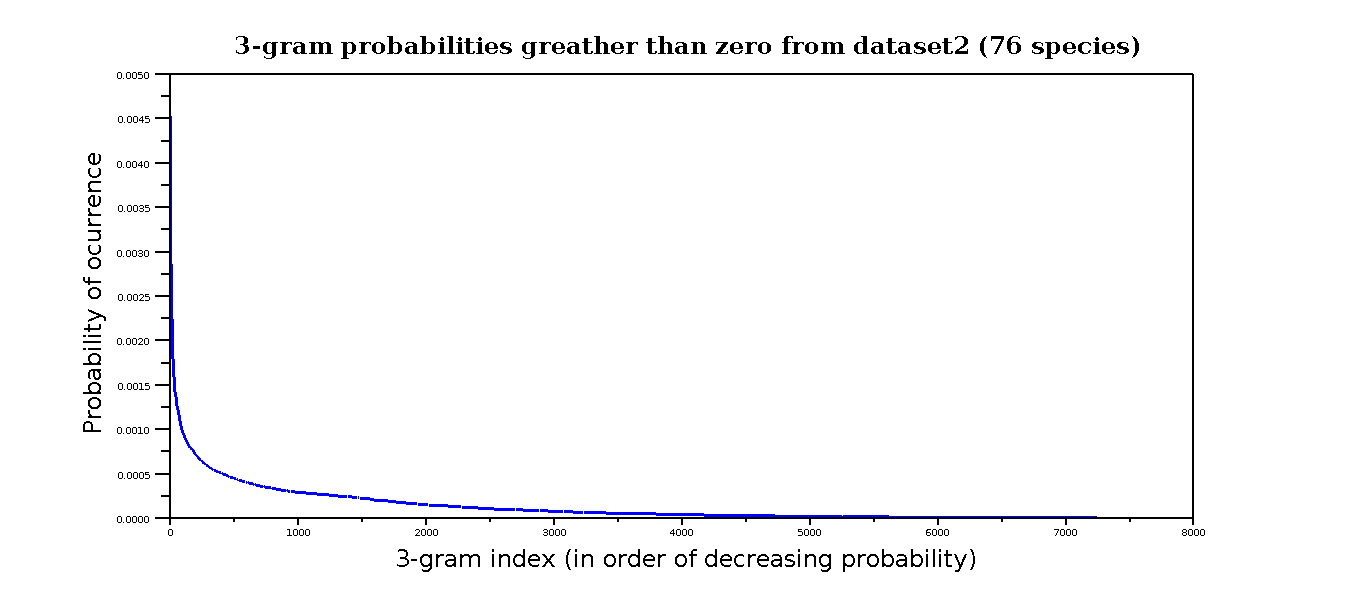

Supplement: Additional file 2 — Scilab algorithms and raw data. In this file, we elaborate on aspects of the algorithms and data used in this research. Algorithms were written in Scilab version "5.2.0.1266391513", scilab-5.2.1. [file 1471-2164-12-S4-S11-S2.zip › SVD_for_improved_taxonomic_classification-S2/76species/3gram/dataset2-3grams.png]
