# Supplementary material for: A singular value decomposition approach for improved taxonomic classification of biological sequences
Source: BMC Genomics. 2011 Dec 22;12(Suppl 4):S11. doi: 10.1186/1471-2164-12-S4-S11 (PMC3287580; doi:10.1186/1471-2164-12-S4-S11)

| | Number of species joined by clusters | | | | Linnaean Taxonomy levels in common by clusters | | | | common Linnaean taxonomy levels frequency (cLtlf) by cluster | | | |
|---|---|---|---|---|---|---|---|---|---|---|---|---|
| **Cluster** | **K-means with rank 60** | **SNJ with rank 60** | **K-means with rank 09** | **SNJ with rank 09** | **K-means with rank 60** | **SNJ with rank 60** | **K-means with rank 09** | **SNJ with rank 09** | **K-means with rank 60** | **SNJ with rank 60** | **K-means with rank 09** | **SNJ with rank 09** |
| **1** | 10 | 10 | 10 | 10 | 10 | 10 | 10 | 10 | 100 | 100 | 100 | 100 |
| **2** | 16 | 27 | 14 | 25 | 10 | 9 | 10 | 9 | 160 | 243 | 140 | 225 |
| **3** | 8 | 1 | 4 | 7 | 6 | 13 | 9 | 8 | 48 | 13 | 36 | 56 |
| **4** | 6 | 17 | 10 | 7 | 10 | 8 | 8 | 8 | 60 | 136 | 80 | 56 |
| **5** | 5 | 2 | 4 | 2 | 9 | 11 | 10 | 12 | 45 | 22 | 40 | 24 |
| **6** | 7 | 1 | 4 | 4 | 8 | 13 | 10 | 10 | 56 | 13 | 40 | 40 |
| **7** | 4 | 1 | 4 | 4 | 12 | 13 | 12 | 12 | 48 | 13 | 48 | 48 |
| **8** | 6 | 1 | 9 | 1 | 10 | 13 | 9 | 13 | 60 | 13 | 81 | 13 |
| | | | | | | | | | 577 | 553 | 565 | 562 |

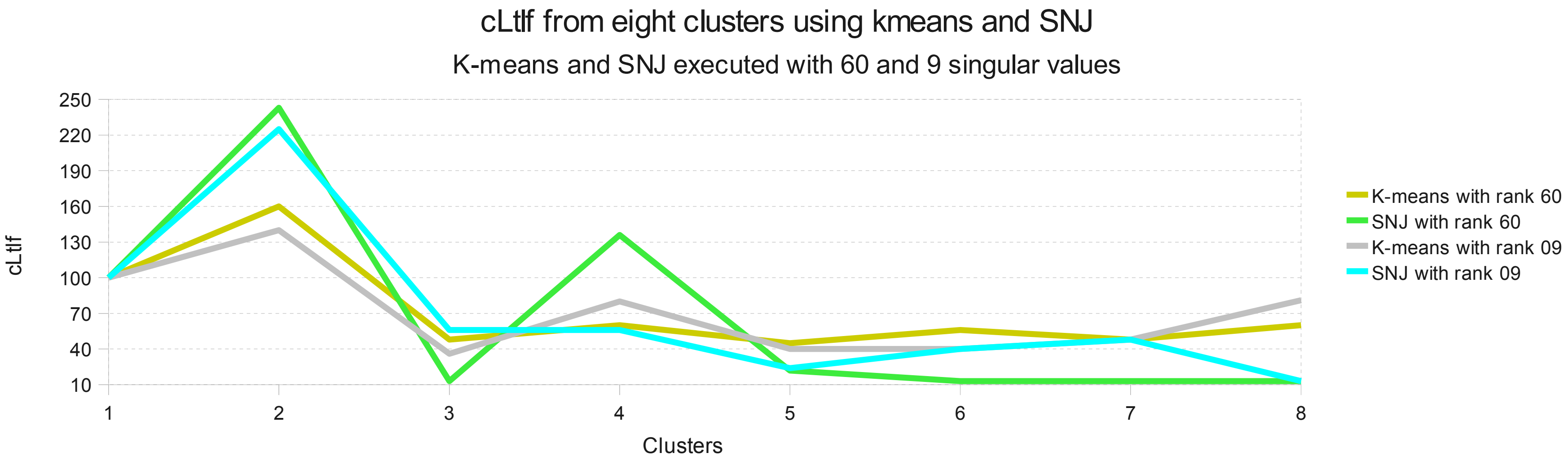

Supplement: Additional file 2 — Scilab algorithms and raw data. In this file, we elaborate on aspects of the algorithms and data used in this research. Algorithms were written in Scilab version "5.2.0.1266391513", scilab-5.2.1. [file 1471-2164-12-S4-S11-S2.zip › SVD_for_improved_taxonomic_classification-S2/76species/60species/kmeans/Compared_Performance.pdf]
