# Supplementary material for: A singular value decomposition approach for improved taxonomic classification of biological sequences
Source: BMC Genomics. 2011 Dec 22;12(Suppl 4):S11. doi: 10.1186/1471-2164-12-S4-S11 (PMC3287580; doi:10.1186/1471-2164-12-S4-S11)
Supplement: Additional file 2 — Scilab algorithms and raw data. In this file, we elaborate on aspects of the algorithms and data used in this research. Algorithms were written in Scilab version "5.2.0.1266391513", scilab-5.2.1. [file 1471-2164-12-S4-S11-S2.zip › SVD_for_improved_taxonomic_classification-S2/76species/53species/Tabelas_Comparativas.pdf]

**Cluster**	**Number of species joined**	**Linnaean Taxonomy levels in common**	**Deepest Linnaean Taxonomy level**
1	10	10	*Carinatae*
2	19	10	*Theria*
3	4	8	*Gnathostomata*
4	6	10	*Neopterygii*
5	3	8	*Gnathostomata*
6	2	12	*Hominidae*
7	4	10	*Elasmobranchii*
8	1	13	*Ornithorhynchus*
9	4	12	*Salmonidae*
10	1	13	*Protopterus*

**Cluster**	**Created with rank 9 from 53 possibilities**	**Created with rank 11 from 58 possibilities**
**1**	Aame Cboy Ccic Cfru Fper Ggal Rame Scam Ssha Vcha	Aame Cboy Ccic Cfru Fper Ggal Rame Scam Ssha Vcha
**2**	Ajam Bmus Bphy Btau Cfam Cpor Csim Easi Ecab Fcat Hamp Hgry Oafe Oari Ocun Pvit Runi Svul Teur	Ajam Bmus Bphy Btau Cfam Cpor Csim Dnov Easi Ecab Fcat Hamp Hgry Oafe Oana Oari Ocun Pvit Runi Sscr Svul Teur
**3**	Amis Eegr Lcha Porn	Amis Eegr Lcha Porn Psub
**4**	Caur Ccar Clac Drer Gmor Poli	Caur Ccar Clac Drer Gmor Poli Salp Ssal
**5**	Cpic Lafr Psub	Cmyd Cpic
**6**	Hsap Ppya	Dvir Mrob
**7**	Mman Rrad Saca Scan	Hsap Ppya
**8**	Oana	Mman Rrad Saca Scan
**9**	Omyk Salp Sfon Ssal	Omyk Sfon
**10**	Pdol	Pdol

Cluster	Number of Species joined		Linnaean Taxonomy levels in common		Deepest Linnaean Taxonomy level	
	Rank 9 from 53	Rank 11 from 58	Rank 9 from 53	Rank 11 from 58	Rank 9 from 53	Rank 11 from 58
**1**	10	10	10	10	*Carinatae*	*Carinatae*
**2**	19	22	10	9	*Theria*	*Mammalia*
**3**	4	5	8	8	*Gnathostomata*	*Gnathostomata*
**4**	6	8	10	10	*Neopterygii*	*Neopterygii*
**5**	3	2	8	11	*Gnathostomata*	*Testudines*
**6**	2	2	12	12	*Hominidae*	*Hominidae*
**7**	4	4	10	10	*Elasmobranchii*	*Elasmobranchii*
**8**	1	2	13	10	*Ornithorhynchus*	*Theria*
**9**	4	2	12	12	*Salmonidae*	*Salmonidae*
**10**	1	1	13	13	*Protopterus*	*Protopterus*
